# From Dysbiosis to Symbiosis: Using the Gut Microbiome to Improve Cancer Outcomes

**DOI:** 10.3390/medsci14060669

**Published:** 2026-10-13

**Authors:** Somia A. Nassar

**Affiliations:** Department of Medical Laboratory Sciences, College of Applied Medical Sciences, Prince Sattam bin Abdulaziz University, Alkharj 11942, Saudi Arabia; s.salem@psau.edu.sa; Tel.: +966-500-938-476

**Keywords:** gut microbiome, dysbiosis, cancer immunotherapy, chemotherapy, fecal microbiota transplantation, microbial metabolites, tumorigenesis, microbiome-based biomarkers, engineered bacteria

## Abstract

The gut microbiome has emerged as an important regulator of cancer biology, influencing carcinogenesis, treatment response, and therapy-related toxicity. This narrative review synthesizes current evidence on the mechanistic roles of the gut microbiota in gastrointestinal (GI) cancers and evaluates emerging microbiome-based strategies for improving cancer outcomes. Dysbiosis—characterized by altered microbial diversity, depletion of beneficial functions, and enrichment of disease-associated taxa—may contribute to tumorigenesis through interconnected mechanisms involving chronic inflammation, genotoxicity, metabolic dysregulation, epigenetic alterations, and immune evasion. Key cancer-associated bacteria, including *Fusobacterium nucleatum*, enterotoxigenic *Bacteroides fragilis* (ETBF), pks^+^*Escherichia coli*, and *Helicobacter pylori*, illustrate the mechanistic diversity of microbiota-associated carcinogenesis. Beyond cancer initiation, the gut microbiome can influence the efficacy and toxicity of anticancer therapies. In immunotherapy, specific microbial taxa and metabolites modulate antitumor immunity and immune checkpoint inhibitor (ICI) responses, while antibiotic-associated dysbiosis has been linked to poorer outcomes. In chemotherapy, microbial metabolism and microbiota-derived metabolites can alter drug efficacy and toxicity, including modulation of 5-fluorouracil (5-FU) activity. Microbiome-based biomarkers show promise for cancer detection and treatment prediction, with fecal microbial signatures, including *F. nucleatum*, potentially improving colorectal cancer (CRC) screening when incorporated into multimarker strategies. Therapeutic approaches aimed at restoring microbial symbiosis include fecal microbiota transplantation (FMT), which has shown preliminary potential to restore ICI responsiveness in treatment-refractory melanoma, and probiotic interventions, although clinical evidence remains limited. Emerging engineered microbial therapeutics, including tumor-targeting bacteria designed to deliver localized immunostimulatory payloads, are advancing toward clinical translation. Despite substantial progress, important challenges remain, including methodological heterogeneity, lack of standardized protocols, confounding factors, and unresolved safety concerns. Personalized strategies integrating microbial features with host characteristics and treatment context may ultimately enable more effective microbiome-based approaches to improve cancer outcomes.

## 1. Introduction

In 2022, the five major GI cancers accounted for 4,783,391 new cases and 3,235,719 deaths worldwide, representing 23.9% and 33.2% of all new cancer cases and deaths, respectively [1]. CRC ranked third in incidence and second in cancer mortality, while gastric cancer ranked fifth in global incidence [1,2]. Multi-omics analysis of colon cancer patients has identified associations between tumor-associated gut microbiota and host transcriptomic profiles, providing insights into microbiota–host interactions in CRC biology [3].

The human gut microbiota comprises diverse microbial communities, with approximately 500–1000 bacterial species predominantly belonging to the phyla Firmicutes, Bacteroidetes, Actinobacteria, and Proteobacteria [4]. These microbial communities contribute to metabolic and immune functions and interact extensively with the host immune system. Gut microbiota and their metabolites can influence systemic antitumor immunity, including cytotoxic CD8^+^ T-cell function and responses to cancer therapy [5,6,7]. The gut microbiota produces bioactive metabolites such as short-chain fatty acids (SCFAs), which can modulate host immune and antitumor responses [5].

Dysbiosis, referring to disruption of microbial composition or function, may involve altered microbial diversity, depletion of commensal taxa, and expansion of potentially pathogenic microorganisms [8]. Dysbiosis can be influenced by dietary patterns and underlying disease, among other host and environmental factors [8,9,10]. Dysbiosis and cancer-associated pathobionts have been implicated in carcinogenesis through interconnected mechanisms involving chronic inflammation, epithelial injury and genotoxic stress, immune dysregulation, and oncogenic signaling pathways [11,12,13].

Beyond cancer initiation, accumulating clinical and translational evidence links gut microbial composition to ICI efficacy. Greater microbial diversity and enrichment of specific metabolically active taxa have been associated with more favorable ICI responses, whereas antibiotic exposure around ICI initiation has been associated with poorer outcomes [14]. Mechanistic studies further support a role for the microbiota in antitumor immunity: microbial metabolites such as butyrate can enhance cytotoxic CD8^+^ T-cell function, while specific bacterial taxa can promote dendritic-cell activation and migration to tumors, thereby enhancing responses to programmed cell death protein 1 (PD-1) blockade [5,6]. The gut microbiota can also influence chemotherapy efficacy and treatment-related toxicity through microbial metabolites and interactions with host metabolic and immune pathways [15,16,17]. These findings support investigation of strategies to restore or modulate microbial homeostasis as potential approaches to improve cancer treatment outcomes.

Scope and focus. This article is a narrative review designed to synthesize mechanistic, translational, and clinical evidence on the role of the gut microbiome in cancer outcomes. It is not intended as a systematic review or meta-analysis but as a critical, mechanism-informed synthesis integrating diverse evidence into a coherent translational framework. The review focuses primarily on GI malignancies, particularly colorectal, pancreatic, and gastric cancers, where microbiome–tumor interactions have been extensively investigated [13,16,17]. However, we deliberately include comparative evidence from melanoma and lung cancer because these settings provide clinically informative evidence linking the gut microbiome to immune checkpoint blockade and illustrate how microbiome–immunotherapy associations may vary across tumor types. This broader perspective allows comparison of mechanisms that may be shared with, or differ from, those observed in GI malignancies [18,19]. Evidence from non-GI tumors is included to illustrate conserved or context-dependent mechanisms relevant to GI oncology and is not intended to imply direct interchangeability across tumor types. Throughout the review, we indicate whether findings are GI-specific, derived from comparative non-GI evidence, or of uncertain generalizability to GI tumors.

A critical note on sampling compartments. A central premise of this review is that microbiome findings should be interpreted according to their anatomical and biological context rather than assumed to be interchangeable across sampling compartments. In colorectal and gastric cancers, tumor-associated bacteria can occupy specific cellular or stromal niches and interact with the tumor microenvironment in ways that may not be captured by faecal profiling alone [20,21,22]. Thus, faecal, mucosal, oral, and intratumoral findings should not be extrapolated across compartments without appropriate comparative or mechanistic evidence. This distinction is particularly relevant for *F. nucleatum*, which has been associated with CRC and treatment-related phenotypes, while evidence from faecal, oral, and tumor-associated samples should be interpreted according to its sampling context [23,24]. The principal compartments, sampling methods, and interpretive caveats are summarized in Table 1.

Review methodology. This narrative review was informed by searches of PubMed/MEDLINE, Scopus, Web of Science, and Google Scholar, with Google Scholar used as a supplementary source. Studies published from January 2000 to March 2026 was considered, with particular emphasis on recent evidence from 2015 onward while retaining earlier landmark studies providing foundational evidence. Search terms combined concepts related to the microbiome, cancer, carcinogenic mechanisms, treatment, biomarkers, and microbiome-based therapeutic strategies, including gut microbiome, dysbiosis, microbial metabolites, carcinogenesis, immune modulation, immunotherapy, chemotherapy, biomarkers, FMT, probiotics, and prebiotics. Studies were selected based on mechanistic and clinical relevance, methodological quality, recency, and landmark status, including preclinical, observational, translational, and interventional studies. Studies with limited relevance to microbiome–cancer interactions or lacking substantive mechanistic or clinical relevance were generally excluded, while relevant guidelines and authoritative sources were retained. Evidence from non-GI cancers was included when it provided clinically or mechanistically relevant insights applicable to GI oncology. As a narrative review, no formal systematic-review risk-of-bias assessment was performed; instead, evidence was interpreted according to study design and strength, with preclinical, observational, and interventional findings clearly distinguished to limit causal over interpretation.

How this review extends beyond existing reviews. Several reviews have examined microbiome–cancer interactions, but this review emphasizes integration, context, compartment, and therapeutic distinction. First, it connects mechanistic pathways with clinically relevant outcomes to provide a translational perspective. Second, it highlights strain-, host-, and tumor-specific effects and considers major sources of observational confounding, including diet and antibiotic exposure. Third, it explicitly distinguishes faecal, mucosa-associated, oral, and intratumoral compartments, reducing unsupported extrapolation across sampling contexts. Fourth, it separates microbiome-modulating strategies—including FMT, probiotics, dietary interventions, and defined microbial consortia—from engineered tumor-targeting bacteria, which use microbial platforms for tumor delivery or local immune activation rather than restoration of intestinal symbiosis. These therapeutic paradigms are therefore discussed separately. Overall, the review provides a mechanism-informed and clinically contextualized synthesis, distinguishing established evidence from emerging or uncertain findings and identifying priorities for future validation.

Scope and aims. This review summarizes the molecular mechanisms linking the gut microbiome to cancer development and treatment response, examines its influence on anticancer therapy efficacy and toxicity, and discusses emerging microbiome-based therapeutic strategies. Specifically, the objectives are to: (1) synthesize mechanisms linking the gut microbiome to carcinogenesis and cancer progression, with attention to sampling compartments; (2) examine microbiome influences on treatment response, toxicity, and resistance across GI and non-GI cancers; (3) evaluate emerging microbiome-based biomarkers for diagnosis, prognosis, and prediction of treatment response; (4) critically appraise microbiome-modulating interventions and engineered microbial therapeutics, distinguishing their mechanisms and clinical status; and (5) identify key evidence gaps and priorities for future research in microbiome-based oncology.

## 2. The Gut Microbiome in Carcinogenesis: Mechanisms of Dysbiosis-Driven Tumorigenesis

Gut dysbiosis can contribute to GI carcinogenesis through interconnected inflammatory, metabolic, genotoxic, epigenetic, and immune-regulatory mechanisms [11,12,13,25]. These processes can interact to promote epithelial injury, genomic instability, immune dysregulation, tumor initiation, progression, and, in some settings, treatment resistance [26,27]. The following sections integrate mechanistic evidence while distinguishing established findings from context-dependent and predominantly preclinical observations.

### 2.1. Pro-Inflammatory Mechanisms and Chronic Inflammatory Signaling

Persistent microbial imbalance can promote mucosal inflammation, oxidative and genotoxic stress, epithelial injury, and immune dysregulation. These responses can alter oncogenic and immune-regulatory pathways that support GI tumor development and progression [11,12,13,25].

ETBF promotes mucosal inflammation through *Bacteroides fragilis* toxin (BFT) mediated activation of NF-κB and STAT3 signaling [11,12]. BFT also rewires epithelial metabolism toward glycolysis, increasing lactate and oxygen availability and supporting bacterial persistence [11]. ETBF-induced IL-17 responses can promote protumoral myeloid-derived suppressor cell (MDSC) accumulation and an immunosuppressive tumor microenvironment [28].

*F. nucleatum* provides another mechanistic example. It can invade cancer-associated fibroblasts (CAFs), inducing an inflammatory phenotype marked by increased IL-6, IL-8, CXCL1, and reactive oxygen species (ROS), thereby enhancing CRC-cell invasion [20]. Similarly, pks^+^*E. coli* and ETBF can induce epithelial injury, DNA-damage responses, and inflammatory transcriptional changes, linking microbial exposure to genotoxic stress and tumor-promoting inflammation [12].

Cytokine signaling provides an important link between microbial inflammation and tumor progression. IL-17 can promote epithelial hyperplasia in ETBF-associated colorectal tumorigenesis, while inflammatory mediators produced by *F. nucleatum*-exposed CAFs can support CRC-cell invasion [11,20]. These effects are context-dependent and may vary according to tumor type, tissue compartment, bacterial strain, and host immune state.

### 2.2. Metabolic Dysregulation and Microbial Metabolites

Dysbiosis can alter the production and availability of microbial metabolites that influence epithelial integrity, host metabolism, inflammatory signaling, and antitumor immunity. The principal metabolite classes implicated in GI carcinogenesis are summarized in Table 2.

Changes in SCFA-producing microbial communities may modify the local availability of metabolites such as butyrate, with potential consequences for intestinal epithelial and inflammatory responses [8]. Butyrate and propionate also have direct epigenetic effects: they can be incorporated into histone lysine acylation marks, alter chromatin accessibility, and influence gene expression in colorectal and intestinal models [29].

Altered microbial bile-acid metabolism can increase the availability of secondary bile acids, including deoxycholic acid (DCA), which can promote colonic epithelial proliferation and tumorigenesis. Recent experimental work showed that a Western diet increased bacterial 7α-dehydroxylation and DCA production and promoted CRC-associated epithelial proliferation in porcine and gnotobiotic mouse models [10].

Microbial tryptophan metabolism generates indole derivatives that can act through the aryl hydrocarbon receptor (AhR) and influence immune regulation [29]. Their biological effects are context-dependent and may vary according to the cellular and tissue environment in which the metabolites are produced or encountered.

Importantly, microbial metabolites may have different effects depending on the biological context. Butyrate, for example, is generally associated with colonic epithelial homeostasis but has also been associated with reduced anti-cytotoxic T-lymphocyte-associated protein 4 (CTLA-4) efficacy when circulating levels are elevated [30]. Thus, microbial abundance alone may not accurately reflect the functional or systemic effects of microbial metabolites [14,30].

### 2.3. Genotoxicity and Genomic Instability

Certain CRC-associated pathobionts can promote genotoxic stress through bacterial virulence factors and host DNA-damage responses. ETBF and pks^+^*E. coli* induce DNA-damage signaling, epithelial disruption, and pro-inflammatory responses in human colon organoids, linking microbial exposure to epithelial DNA damage and tumor-promoting inflammation [12].

Colibactin-producing pks^+^*E. coli* induces DNA damage, including double-strand breaks and replication-associated lesions. Colibactin forms DNA adducts and interstrand cross-links that can generate characteristic mutational signatures associated with CRC [12,31].

Cytolethal distending toxin (CDT), produced by several pathogenic bacterial species, induces DNA damage and DNA-damage responses, resulting in cell-cycle disturbances, altered ploidy, and genomic instability. Experimental evidence further implicates YAP/TAZ–TEAD signaling in cellular responses to CDT- and colibactin-induced DNA damage [32].

*F. nucleatum* may contribute to oxidative stress and cellular responses relevant to DNA damage through inflammatory signaling and ROS production [20]. This differs from the more direct genotoxic effects attributed to colibactin and CDT.

The magnitude and biological effects of bacterial genotoxicity may vary with bacterial strain, host-cell context, and tissue environment. Thus, bacterial taxonomy alone may not reliably indicate genotoxic risk [12,31].

### 2.4. Epigenetic Modifications

Gut microorganisms and their metabolites can influence host gene expression through epigenetic mechanisms, including DNA methylation and histone modifications [33,34].

*F. nucleatum* infection can induce transcriptomic and epigenomic alterations in CRC cells, including changes in DNA methylation and other regulatory features of the host epigenome [35]. Microbial metabolites can also directly affect chromatin regulation. SCFAs such as butyrate and propionate can modify histone lysine residues and influence chromatin accessibility and gene expression [36]. In addition, specific microbiota–RNA interactions can alter microRNA and long non-coding RNA pathways involved in colorectal tumorigenesis and treatment response [24,37].

These effects are highly context-dependent and may vary according to cell type, tumor context, microbial composition, and metabolite availability [33,36].

Importantly, much of the current mechanistic evidence for microbiota-associated epigenetic regulation comes from cell and animal models, whereas direct causal evidence in human tumors in vivo remains limited.

### 2.5. Immune Suppression and Immune Evasion

Dysbiosis can remodel the tumor immune microenvironment by altering immune-cell recruitment and inflammatory signaling. Microbiota-associated changes can promote immunosuppressive myeloid populations, IL-17-dependent responses, and programmed death-ligand 1 (PD-L1) expression [22,25]. In CRC, *F. nucleatum* can also induce an inflammatory CAF phenotype, increasing cytokine and ROS production and promoting tumor-cell invasion [20].

In CRC models, the *F. nucleatum* Fap2 adhesin can bind TIGIT on NK and T cells and inhibit their cytotoxic activity [38]. *Peptostreptococcus anaerobius* can promote an immunosuppressive tumor microenvironment through integrin α2β1/NF-κB/CXCL1 signaling and MDSC recruitment [27].

Recent experimental evidence indicates that gut dysbiosis can promote immunosuppressive CD8^+^ T-cell phenotypes, including increased CTLA-4 expression. In CRC, enrichment of *Bacteroides* and *Phocaeicola* was associated with increased CTLA-4^+^PD-1^+^ CD8^+^ T-cell populations and impaired T-cell effector function, while FMT reduced CD8^+^CTLA-4^+^ T-cell formation and promoted CTL activation [21].

In gastric cancer, *H. pylori* can contribute to an immunosuppressive microenvironment through M2-like macrophage polarization [39], impairment of dendritic-cell maturation through IL-10/STAT3-dependent mechanisms [40], and induction of PD-L1 expression through CagA-dependent Sonic Hedgehog signaling [41].

Microbial communities may also influence immune regulation through Treg and Th17 differentiation and antimicrobial responses. For example, *F. nucleatum* can promote dendritic-cell activation and disrupt the Th17/Treg balance in intestinal inflammation [42]. Altered antimicrobial-peptide responses may contribute to dysbiosis and impaired epithelial-barrier function [43], while tumor-associated microbial communities can promote IL-17-dependent B-cell responses associated with tumor progression [25].

The immunological effects of individual taxa are context-dependent and vary according to bacterial strain, tissue compartment, tumor type, host immune state, and treatment context. For example, intratumoral *F. nucleatum* has been reported to recruit tumor-associated neutrophils in gastric cancer [22], whereas *B. fragilis* polysaccharide A (PSA) has immunomodulatory effects in experimental models, including induction of regulatory T-cell and IL-10 responses [44]. These experimental findings do not establish clinical benefit from PSA-based microbiome modulation or ICI therapy.

### 2.6. Integration of Mechanisms

The mechanisms described above are interconnected rather than independent. Dysbiosis can alter microbial metabolism and epithelial-barrier function, increasing exposure to inflammatory mediators and bacterial genotoxins. These changes may promote DNA damage, epigenetic alterations, chronic inflammation, and immune dysfunction, which can collectively contribute to tumor initiation and progression [11,12,25,35].

However, the existence of these interconnected pathways does not imply that dysbiosis is sufficient to cause cancer in every setting. Their relative contribution is likely to depend on microbial strain and function, host genetics, diet, tissue compartment, tumor type, and treatment context. The principal mechanisms through which gut microbial dysbiosis and specific microbial taxa contribute to gastrointestinal carcinogenesis, including effects on inflammation, epithelial signaling, genomic stability, and the tumor microenvironment, are summarized in Table 3.

### 2.7. Key Bacteria and Their Principal Mechanisms

Specific cancer-associated bacteria, including *Fusobacterium* spp., *B. fragilis*, *E. coli*, and *H. pylori*, have been implicated in GI tumorigenesis through diverse effects on epithelial integrity, inflammation, genotoxic stress, immune regulation, and host signaling [8,12]. Their principal mechanisms and associated GI cancers are summarized in Table 4.

## 3. The Gut Microbiome as a Modulator of Cancer Therapy Outcomes

### 3.1. Immunotherapy: The Microbiome–ICI Axis

The gut microbiome can influence the efficacy and toxicity of cancer immunotherapy through effects on systemic immunity, the tumor immune microenvironment, and microbial metabolite production. These interactions can affect T-cell activation and differentiation, antigen presentation, immune-cell recruitment, inflammatory signaling, and responses to ICIs, particularly PD-1/PD-L1 blockade [7,48,49].

Recent single-cell transcriptomic analyses in preclinical models showed that an intact gut microbiota enhanced ICI efficacy by increasing effector and memory CD8^+^ and CD4^+^ T-cell populations, reducing CD8^+^ T-cell exhaustion, and promoting reprogramming of SPP1^+^protumoral tumor-associated macrophages toward CD74^+^ antigen-presenting states [7]. These findings support a microbiota–immune axis extending beyond individual bacterial taxa to include coordinated changes in immune-cell states and cellular interactions within the tumor microenvironment.

#### 3.1.1. Mechanisms of Microbiome–ICI Interaction

##### Modulation of Immune Responses

The gut microbiota contributes to systemic immune regulation and can influence the magnitude and quality of antitumor responses during ICI therapy [7,48]. Clinical studies have associated specific microbial profiles with ICI responsiveness. For example, in patients with advanced non-small-cell lung cancer (NSCLC), enrichment of Ruminococcaceae UCG-013 and *Agathobacter* was associated with objective response and longer progression-free or overall survival [50].

Microbial signals can regulate dendritic-cell function, antigen presentation, and T-cell activation. In experimental models, polysaccharide A from *B. fragilis* promotes immunoregulatory signaling and IL-10-producing regulatory T-cell responses through TLR2/TLR1- and Dectin-1-dependent pathways [44]. Microbial metabolites can also modify ICI activity. In melanoma models and patients receiving CTLA-4 blockade, elevated circulating butyrate and propionate were associated with reduced treatment efficacy and altered dendritic-cell and T-cell responses [30].

*Akkermansia muciniphila* provides another example of a taxon associated with ICI response. In a prospective NSCLC cohort, higher baseline fecal *A. muciniphila* abundance was associated with higher objective response rates and longer overall survival, while experimental supplementation enhanced anti-PD-1 efficacy and promoted restoration of CD8^+^ T-cell effector states [19,51].

The effects of microbiome perturbation are not uniformly beneficial or detrimental. Antibiotic exposure before or during ICI therapy has been associated with poorer clinical outcomes in several cohorts, although the magnitude and independence of this association vary across treatment settings [14,52]. Experimental evidence further indicates that microbiome disruption can impair antitumor immunity during checkpoint blockade. However, clinical antibiotic exposure may also reflect infection, comorbidity, disease severity, or other prognostic factors and therefore should not be interpreted as definitive evidence of causality [14,50].

#### 3.1.2. Remodeling the Tumor Microenvironment

The gut microbiota can shape the tumor immune microenvironment through coordinated effects on tumor-infiltrating lymphocytes, macrophages, antigen-presenting cells, and microbial metabolites [7,48]. Single-cell analyses have shown that microbiota–ICI interactions can increase intratumoral CD8^+^, CD4^+^, and γδ T-cell populations while promoting reprogramming of SPP1^+^protumoral macrophages toward CD74^+^ antigen-presenting states [7].

Microbial metabolites provide an additional link between intestinal bacteria and immune-cell function. Bile-acid metabolism, for example, can regulate antitumor CD8^+^ T-cell activity. DCA, a secondary bile acid generated by microbial transformation of primary bile acids, suppresses CD8^+^ T-cell effector function through inhibition of PMCA/Ca^2+^–NFAT2 signaling. In experimental CRC models, bacteria harboring DCA-biosynthetic pathways promoted tumor growth, whereas disruption of microbial DCA metabolism or bacteriophage targeting of DCA-producing bacteria restored antitumor immune activity [53].

Together, these findings indicate that microbiome-associated immune effects involve both systemic immune regulation and local remodeling of the tumor microenvironment rather than a single bacterial or immune-cell pathway.

#### 3.1.3. Key Microbial Metabolites in Immunotherapy

Microbial metabolites provide an important interface between the gut microbiota and host immunity. SCFAs, bile acids, tryptophan-derived metabolites, and trimethylamine N-oxide (TMAO) can influence immune-cell function and antitumor responses, but their effects depend on the specific metabolite, concentration and site of exposure, tumor type, and therapeutic context [29,36,53,54]. The principal metabolite classes implicated in antitumor immunity, together with their microbial sources, mechanisms of action, and context-dependent effects, are summarized in Table 5.

Clinical and translational studies indicate that both microbial composition and microbial metabolic activity may contribute to ICI outcomes. In selected ICI-treated cohorts, enrichment of SCFA-producing bacteria has been associated with favorable outcomes, whereas elevated circulating butyrate and propionate have been associated with reduced CTLA-4 blockade efficacy in melanoma [30,50]. Similarly, indole-3-aldehyde and TMAO have been implicated in experimental models of checkpoint-blockade response [29,54]. These apparently contrasting observations emphasize the importance of distinguishing microbial abundance from functional activity and from circulating metabolite concentrations.

Dietary factors may further modify microbiome-mediated immune responses. Experimental studies have shown that high-fat dietary patterns can alter the microbiome and immune pathways relevant to cancer progression [9]. Recent experimental and translational evidence also indicates that sucralose consumption can alter microbial metabolism, increase microbial arginine degradation, impair T-cell function, and reduce anti-PD-1 responses; higher sucralose intake was additionally associated with poorer outcomes in patients receiving anti-PD-1-based therapy [49].

A major limitation of the human literature is its predominantly observational nature. Associations between microbiome composition, microbial metabolites, and ICI outcomes may be influenced by diet, medications, antibiotic exposure, disease severity, treatment characteristics, geographic variation, and baseline microbiome differences [30,49,50]. Accordingly, associations between individual microorganisms or metabolites and ICI efficacy should not automatically be interpreted as evidence of causality.

#### 3.1.4. Bacterial Strains, FMT, and Antibiotic Effects

##### Specific Bacterial Strains Associated with ICI Efficacy

Several bacterial taxa or strains have been associated with ICI responses in clinical and experimental studies, although no universal set of beneficial bacteria has been established [6,29,50].

The strain YB328 (*Hominenteromicrobium mulieris*) was enriched in patients responding to PD-1 blockade and enhanced anti-PD-1 efficacy in experimental models. YB328 promoted differentiation and activation of CD103^+^CD11b^−^ conventional dendritic cells, which migrated from the gut to tumor-draining lymph nodes and the tumor microenvironment, where they promoted tumor-specific CD8^+^ T-cell responses [6]. Importantly, this evidence derives from PD-1 blockade models and clinical cohorts rather than radiotherapy.

*L. reuteri* similarly enhanced checkpoint blockade in experimental models through its tryptophan metabolite indole-3-aldehyde, which promotes antitumor CD8^+^ T-cell responses [29]. *B. fragilis* polysaccharide A modulates immune responses through TLR2/TLR1- and Dectin-1-dependent signaling and promotes IL-10-producing regulatory T-cell responses, although direct evidence of clinical ICI benefit remains limited [44]. Members of the Ruminococcaceae family have also been associated with favorable ICI outcomes in selected clinical cohorts [50].

Increasing evidence suggests that ecological and functional properties of the microbiome may provide more informative predictors than taxonomic abundance alone [56].

##### Fecal Microbiota Transplantation and ICI Response

FMT provides experimental and early clinical evidence that the gut microbiome can influence sensitivity to immune checkpoint blockade. In preclinical models, transfer of microbiota associated with immunotherapy responsiveness enhanced antitumor immunity and anti-PD-1 responses [48]. Early clinical studies in metastatic melanoma refractory to anti-PD-1 therapy showed that responder-derived FMT combined with anti-PD-1 reinduction produced objective responses or clinical benefit in a subset of patients [57,58].

These studies involved small populations and selected clinical settings, limiting generalizability to other tumor types and broader patient populations. FMT therefore remains investigational, with donor selection, treatment protocols, durability, safety, and efficacy requiring validation in larger controlled studies [57,58].

##### Antibiotic Exposure and ICI Outcomes

Antibiotic exposure can perturb the gut microbiome and has been associated with poorer outcomes during ICI therapy, although the magnitude of this association varies among studies [14,52]. Experimental evidence supports a causal contribution of microbiome disruption to impaired antitumor immunity during checkpoint blockade, but clinical antibiotic exposure is subject to substantial confounding [49]. Antibiotic use should therefore be considered a potential modifier of ICI responsiveness rather than an established independent cause of treatment resistance.

### 3.2. Chemotherapy: Efficacy, Response, and Toxicity

The gut microbiota can influence chemotherapy efficacy, resistance, and toxicity through microbial metabolism, bioactive metabolites, immune modulation, and direct interactions with anticancer drugs [15,16,17,59].

Recent experimental work illustrates the capacity of microbial metabolites to alter chemotherapy activity. The microbiota-derived metabolite 2-methylisocitrate enhanced 5-FU activity through inhibition of mitochondrial isocitrate dehydrogenase, disruption of nucleotide homeostasis, and induction of DNA damage [15]. Conversely, enrichment of the bacterial preTA operon during fluoropyrimidine treatment was associated with reduced treatment toxicity, while preTA-expressing bacteria experimentally inactivated 5-FU [16].

Microbiome composition has also been associated with chemotherapy outcomes. In patients with extensive-stage small-cell lung cancer receiving chemotherapy combined with immunotherapy, differences in microbial composition and metabolites were observed between patients with longer and shorter progression-free survival (PFS), although these observational findings do not establish causality [59]. In pancreatic cancer, *E. coli*, *E. faecalis*, and *F. nucleatum* were associated with higher chemotherapy-resistance risk, whereas *Akkermansia* and *Bifidobacterium* were associated with lower risk in a retrospective cohort [17].

#### 3.2.1. Drug Resistance Mechanisms

Gut microorganisms can modify chemotherapy responses through direct drug metabolism, production of bioactive metabolites, modulation of tumor-cell signaling, alteration of host immunity, and regulation of cellular survival pathways [5,15,16,24].

*F. nucleatum* is a well-characterized contributor to CRC chemoresistance. It promotes chemoresistance through TLR4/MYD88-associated autophagy pathways and, in experimental models, through inhibition of ferroptosis involving the E-cadherin/β-catenin/TCF4/GPX4 axis [24,46]. Tumor-associated *F. nucleatum* abundance has also been associated with CRC relapse, while experimental models demonstrate its role in oxaliplatin resistance [46].

These findings illustrate that microbiota–drug interactions can either enhance or impair chemotherapy responses depending on the microbial species or metabolite and the treatment context. Butyrate, for example, enhanced oxaliplatin efficacy in experimental models through an ID2-dependent IL-12 signaling mechanism involving CD8^+^ T-cell function [5]. In contrast, *F. nucleatum* can promote chemoresistance, while bacterial preTA activity can inactivate 5-FU and modify fluoropyrimidine toxicity [16,24].

#### 3.2.2. Toxicity Mitigation

##### Chemotherapy-Induced Gastrointestinal Toxicity

Chemotherapy commonly causes GI toxicity, including diarrhea, mucositis, nausea, vomiting, and disruption of intestinal epithelial barrier integrity [60,61]. Approximately 40% of patients receiving standard-dose chemotherapy may develop GI-related toxicities, with reported rates reaching 60–100% in some high-dose chemotherapy settings [60].

Microbial metabolism can modify this toxicity. Enrichment of the bacterial preTA operon during oral fluoropyrimidine treatment was associated with reduced fluoropyrimidine toxicity in patients, while preTA-expressing bacteria experimentally inactivated 5-FU and ameliorated fluoropyrimidine-induced toxicity [16].

##### Probiotic Interventions

Probiotic administration has been investigated as a strategy for reducing chemotherapy-associated GI complications. In a randomized, single-blind, placebo-controlled study of 100 patients with CRC receiving postoperative chemotherapy, a four-strain probiotic formulation containing *B. infantis*, *L. acidophilus*, *E. faecalis*, and *B. cereus* attenuated GI complications, partially restored chemotherapy-associated changes in gut microbial composition, and increased fecal acetate, butyrate, and propionate concentrations [61]. These findings should not be generalized to all probiotic formulations or cancer populations because they were obtained with a specific formulation in postoperative CRC patients receiving chemotherapy.

In a separate genetically engineered mouse model of pancreatic ductal adenocarcinoma, combined *Lactobacillus paracasei* GMNL-133 and *Lactobacillus reuteri* GMNL-89 enhanced the antitumor effects of gemcitabine, reflected by reduced PanIN severity and lower proliferation and epithelial–mesenchymal transition markers. The combination was also associated with lower serum alanine aminotransferase and aspartate aminotransferase levels [62].

Preclinical evidence also suggests that combined *L. reuteri* and *Clostridium butyricum* may attenuate cisplatin-induced renal injury and inflammation by partially restoring gut microbiota composition, increasing butyrate production, and modulating gut–kidney axis-related pathways [63].

##### Microbial Drug Metabolism and Agent-Specific Effects

The examples above illustrate the broader pharmacomicrobiomic concept that bacterial enzymes and microbial metabolites can alter anticancer-drug activity and toxicity [15,16,64].

For 5-FU, the bacterial preTA operon can promote drug inactivation and reduce fluoropyrimidine toxicity, whereas microbiota-derived 2-methylisocitrate can enhance 5-FU activity [15,16].

For oxaliplatin, butyrate can enhance antitumor efficacy through CD8^+^ T-cell activation via an ID2-dependent IL-12 signaling mechanism, whereas *F. nucleatum* promotes CRC chemoresistance through TLR4/MYD88-associated autophagy and ferroptosis inhibition [5,24,46].

For irinotecan, bacterial β-glucuronidases can reactivate SN-38G to cytotoxic SN-38 in the gut, contributing to intestinal mucosal injury and delayed GI toxicity, particularly diarrhea. Targeted inhibition of bacterial β-glucuronidases has therefore been investigated as a predominantly preclinical strategy for reducing irinotecan-associated toxicity [64].

Overall, these examples indicate that microbiome–chemotherapy interactions are drug- and context-dependent and may involve microbial composition, functional activity, metabolites, host immunity, and treatment exposure rather than taxonomic composition alone [15,16,64].

### 3.3. Radiotherapy

The gut microbiome has been associated with radiotherapy response and treatment-related toxicity. Clinical studies have linked microbiome composition or predicted functional characteristics with treatment outcomes and toxicity, whereas preclinical studies provide mechanistic evidence for microbiota-mediated effects on radiotherapy-associated antitumor immunity [65,66,67,68].

Clinical evidence remains predominantly observational. In patients with lung cancer receiving chemoradiotherapy followed by consolidation immunotherapy, gut microbiota characteristics were associated with treatment outcomes and toxicity [66]. Similarly, a prospective observational cohort of patients with locally advanced cervical cancer receiving concurrent chemoradiotherapy identified associations between intestinal flora changes and acute radiation enteritis, particularly diarrhea [67]. In patients with lung cancer brain metastases receiving radiotherapy, microbiome composition and predicted functional characteristics were also associated with treatment response [65].

Preclinical evidence provides additional mechanistic support. In microsatellite-stable CRC models, antibiotic-mediated disruption of the gut microbiota reduced the efficacy of combined radiotherapy and immunotherapy, whereas oral *B. fragilis* or propionate enhanced CD8^+^ T-cell infiltration and treatment responses [68].

Overall, current evidence supports an association between the gut microbiome and radiotherapy response or toxicity, but causal inference remains limited by the predominance of observational clinical studies and the preclinical nature of much of the mechanistic evidence. Prospective, adequately powered interventional studies are needed to determine whether microbiome-directed interventions can safely and reproducibly improve radiotherapy outcomes [66,67,68].

## 4. The Microbiome as a Biomarker: From Diagnosis to Prognosis

Microbiome-based biomarkers are being investigated across the cancer continuum, from early detection and diagnosis to prognosis and treatment-response assessment [3,56]. However, the maturity of evidence varies substantially among these applications. This section distinguishes diagnostic, prognostic, and predictive biomarkers and summarizes reported performance characteristics, including sensitivity, specificity, area under the receiver operating characteristic curve (AUC), confidence intervals, sample size, and validation design, where available. Importantly, case–control discrimination is distinguished from performance in intended screening populations, and internal validation from independent external validation.

### 4.1. Diagnostic Biomarkers

Microbial profiling of non-invasive specimens, particularly stool and saliva, has shown potential for cancer detection. Several studies have evaluated microbial signatures alone or in combination with established screening markers such as fecal immunochemical testing (FIT), with promising diagnostic performance reported for CRC and advanced adenoma [23,69,70,71]. However, most microbiome-based diagnostic biomarkers remain investigational and require validation in larger, prospective, multicenter screening populations. Representative performance characteristics are summarized in Table 6.

#### 4.1.1. Colorectal Cancer (CRC)

*F. nucleatum* is among the most extensively investigated fecal microbial markers for CRC and advanced colorectal neoplasia, particularly when combined with the fecal immunochemical test (FIT) [23,69,71]. In a study comprising 309 participants in the primary cohort and an independent validation cohort of 181 subjects, addition of fecal *F. nucleatum* to FIT increased CRC sensitivity from 73.1% to 92.3% and AUC from 0.86 to 0.95. For advanced adenoma (AA), sensitivity increased from 15.5% to 38.6% and AUC from 0.57 to 0.65, with comparable performance in the independent validation cohort [23]. The increase in CRC sensitivity was accompanied by a reduction in specificity from 98.0% with FIT alone to 93.0% with the combined test, illustrating the trade-off between sensitivity and specificity.

A four-marker fecal panel comprising *F. nucleatum*, *Lachnoclostridium* sp. m3, *Clostridium hathewayi*, and *Bacteroides clarus*, combined with FIT, has also been evaluated for CRC and AA detection. In 676 subjects, including 435 asymptomatic participants, the four-marker panel detected 84.9% of CRC and 38.6% of AAs in the asymptomatic cohort [71]. These findings support microbial markers as potential complements to FIT, although additional positive tests may increase the number of individuals requiring diagnostic colonoscopy.

Another fecal-based model incorporating *F. nucleatum*, *Peptostreptococcus anaerobius*, methylated ITGA4, and FIT achieved an AUC of 0.958 for combined detection of CRC and AA [69]. The model was subsequently evaluated in an independent external validation cohort and a CRC screening cohort, where it retained substantial discriminatory performance. This study illustrates the importance of distinguishing case–control discrimination from evaluation in screening populations and of testing candidate biomarkers outside the original development cohort [69]. The external validation cohort was collected from three institutions, while the screening cohort included participants aged 45–75 years.

Despite these findings, *F. nucleatum* should not be regarded as a validated standalone diagnostic biomarker. Performance may vary according to population, disease stage, specimen characteristics, assay methodology, and analytical thresholds, and the organism is not specific to CRC [69,72]. Current evidence therefore supports its evaluation primarily as a complementary marker alongside established screening approaches such as FIT [69,71].

#### 4.1.2. Other Cancers

Microbiome-based diagnostic approaches have also been investigated in gastric, pancreatic, breast, and lung cancers, although study designs, specimen types, cohort sizes, and analytical methods vary substantially [4,73].

In pancreatic cancer, a 2025 case–control study involving 41 patients and 40 matched controls evaluated salivary *Porphyromonas gingivalis* and *Aggregatibacter actinomycetemcomitans* together with circulating miR-21, miR-155, and diabetes status. The combined model achieved an AUC of 0.878 (95% CI, 0.802–0.955), with 87.8% sensitivity and 78.9% specificity [70]. Its small, single-center case–control design and absence of independent external validation limit conclusions regarding performance in screening populations.

In breast cancer, observational studies have reported differences in fecal bacterial abundance, including reductions in *Lactobacillus* and *Bifidobacterium*, but these associations have not established a clinically validated diagnostic biomarker [4].

Overall, microbial profiling may complement conventional cancer detection strategies, but variation in specimen collection, laboratory processing, analytical pipelines, and study populations limits cross-study comparability. Prospective validation in representative populations, standardized analytical procedures, and demonstration of clinical utility are needed before microbiome-based diagnostic biomarkers can be incorporated into routine screening or diagnosis [69,73].

### 4.2. Prognostic Biomarkers

Prognostic biomarkers are associated with clinical outcomes such as recurrence, progression, or survival within a defined patient population. Predictive biomarkers, in contrast, identify patients whose benefit from a specific treatment differs according to biomarker status. Accordingly, an association between a microbial feature and outcome in a treated cohort should not be interpreted as treatment-specific predictive utility without appropriate comparative evidence.

#### 4.2.1. Intratumoral Microbiome

The intratumoral microbiome has been associated with tumor progression, metastasis, prognosis, and treatment response [22,74,75]. Because microbial composition and function differ among anatomical niches, findings from intratumoral specimens should not be directly extrapolated to fecal or mucosa-associated compartments.

In gastric cancer, intratumoral *F. nucleatum* was enriched in tumors from patients with lymph-node metastasis and associated with poorer progression-free and overall survival. Experimental findings further implicated *F. nucleatum* in tumor progression and immune evasion through recruitment and phenotypic modulation of tumor-associated neutrophils [22].

In CRC, higher tumor-tissue abundance of *F. nucleatum* was associated with relapse and has been linked experimentally to oxaliplatin resistance through inhibition of ferroptosis [46]. These observations support prognostic and treatment-response associations but do not establish differential treatment benefit.

#### 4.2.2. Primary Tumor Microbiomes and Metastatic Risk

In a retrospective cohort of 900 CRC patients, primary-tumor microbiome composition was associated with 5-year metastasis-free survival. Enterobacteriaceae, including *E. coli*, were enriched in distant metastases, while enterotoxigenic *B. fragilis* (ETBF) and pks-positive *E. coli* were more frequently detected in primary tumors from patients who subsequently developed metastases. These findings were independently assessed using 16S rDNA sequencing and pathobiont-specific quantitative PCR [75]. They support the prognostic relevance of the primary-tumor microbiome but do not demonstrate treatment-specific predictive utility.

#### 4.2.3. Pancreatic Cancer: Gut Microbiome-Based Risk Model

In a retrospective cohort of 510 patients with pancreatic cancer receiving chemotherapy, *E. coli*, *E. faecalis*, and *F. nucleatum* were identified as independent risk factors for chemotherapy resistance. A gut microbiota-based nomogram was developed using 357 patients in a training cohort and evaluated in a randomly separated cohort of 153 patients. The AUCs were 0.767 (95% CI, 0.708–0.827) and 0.762 (95% CI, 0.669–0.854), respectively, with sensitivities of 45.5% and 51.1% and specificities of 86.5% and 87.3% [17]. Because the validation cohort was derived from the same study population, this represents internal rather than independent external validation.

Importantly, the nomogram was based on fecal samples and therefore represents a gut microbiome-based model rather than an intratumoral microbiome biomarker [17].

Overall, current evidence supports associations between specific microbial features and prognosis or treatment outcomes, but most studies remain limited by cohort size, observational designs, compartmental heterogeneity, and incomplete external validation [18,72,74,75]. Mechanistic evidence that the microbiome modulates antitumor immunity and treatment response further supports biological plausibility but does not itself establish biomarker predictive utility [48].

### 4.3. Biomarkers Associated with Treatment Response

Microbial features associated with treatment response have been investigated most extensively in ICB. However, response associations within treated populations should be distinguished from validated predictive biomarkers, which require evidence that treatment benefit differs according to biomarker status, ideally through an appropriate treatment comparator.

In gastric cancer, *H. pylori*-positive patients receiving immunotherapy had longer immune-related progression-free and overall survival than *H. pylori*-negative patients, and incorporating *H. pylori* status into a gut microbiome-based model improved prediction of immunotherapy outcomes [74]. Because the analysis involved an immunotherapy-treated cohort without a treatment comparator, these findings represent response-associated evidence rather than demonstrated treatment-specific predictive utility.

In hepatocellular carcinoma (HCC), 80 patients receiving ICI-based therapy were prospectively enrolled, and an 18-species gut bacterial model was developed to distinguish durable from non-durable clinical benefit. The model achieved an AUC of 0.7563 (95% CI, 0.5834–0.9293) in the 31-patient test cohort after LASSO-based development and fivefold cross-validation in 49 patients [72]. The separate test cohort provides internal evaluation, but independent external validation remains lacking.

In advanced melanoma, several gut taxa, including *Agathobaculum butyriciproducens*, *Intestinibacter bartlettii*, and *Lactobacillus gasseri*, were more abundant among patients with PFS of ≥12 months during ICB [18]. These findings identify treatment-associated microbial features but do not establish differential benefit from ICB compared with another treatment.

The pancreatic cancer nomogram described above also predicted chemotherapy resistance, with AUCs of 0.767 and 0.762 in the training and internally validated cohorts, respectively [17]. Its single-institution design and internal validation limit generalizability.

Taken together, baseline microbiome profiles show potential for patient stratification and treatment-response assessment, particularly during immunotherapy. However, most evidence remains exploratory or response-associated, and validated treatment-specific predictive biomarkers remain limited. Independent external validation and prospective clinical-utility studies are needed before microbiome-based biomarkers can guide routine treatment selection.

### 4.4. Overcoming Microbiome-Mediated Resistance

The gut and tumor-associated microbiome may influence cancer therapy through microbial taxa, metabolites, and host–microbiome interactions, with some microbial features promoting resistance and others enhancing therapeutic responses [27,54,76].

In CRC, *P. anaerobius* impaired anti-PD-1 efficacy experimentally through recruitment and immunosuppressive activation of intratumoral myeloid-derived suppressor cells [27]. In pancreatic cancer models, targeted modulation of the intratumoral microbiota with gallium–polyphenol-functionalized *Lactobacillus rhamnosus* GG (LGG@Ga-poly) reduced tumor-associated microbial lipopolysaccharide and enhanced the efficacy of ICB [76]. Microbial metabolites may also affect therapeutic responses; for example, TMAO promoted antitumor immunity and improved immunotherapy efficacy in triple-negative breast cancer models [54].

Microbiome-associated chemoresistance has similarly been described for *F. nucleatum* in CRC through inhibition of ferroptosis, activation of the TLR4/MYD88–microRNA–autophagy pathway, and suppression of chemotherapy-induced pyroptosis through the Hippo/YAP–BCL2 axis (See Section 3.2.1) [24,46,77]. Other resistance-associated taxa, including *E. coli* and *E. faecalis*, are summarized in.

#### Strategies to Overcome Microbiome-Mediated Resistance

Microbiome-directed interventions include FMT, targeted manipulation of intratumoral microbial communities, and approaches that exploit microbial–immune interactions [76,78]. In preclinical pancreatic cancer models, LGG@Ga-poly enhanced PD-L1 blockade [76].

FMT has been investigated clinically, particularly for treatment-refractory ICI-induced colitis [78]. Separate early studies in melanoma have explored FMT to overcome anti-PD-1 resistance, but these findings remain preliminary and should not be conflated with the evidence for FMT in ICI-induced colitis [57,58].

Low-dose intestinal irradiation has also been investigated as a strategy to enhance PD-L1 blockade, with effects involving metabolic and immune host–microbiota interactions [79]. Overall, microbiome-directed strategies remain largely experimental or restricted to selected clinical settings. Prospective controlled trials with standardized microbiome assessment, clinically relevant endpoints, and independent validation are needed before routine therapeutic implementation [78,79].

### 4.5. Limitations and Challenges in Microbiome-Based Biomarker Development

Despite their potential, microbiome-based biomarkers face methodological, biological, and clinical limitations that currently restrict routine implementation [18,73,80].

#### 4.5.1. Heterogeneity of Microbiome Profiling Methods

Differences in sample processing, DNA extraction, sequencing, and analytical workflows can substantially affect estimates of microbial composition and limit reproducibility and cross-study comparability [73,80]. In two independent cohorts, different DNA-extraction methods altered the differential abundance of more than 75% of detected species, illustrating the magnitude of methodological variation [80]. Harmonized workflows and standardized quality-control procedures are therefore important for improving reproducibility and comparability [73,80].

#### 4.5.2. Population Differences and Confounding

Microbiome composition varies across populations and may be influenced by geographic location, diet, lifestyle, and other environmental factors [8]. Treatment regimen, antibiotic and proton-pump inhibitor exposure, previous anticancer therapy, demographic characteristics, and other clinical factors may also modify microbiome–outcome associations [18]. Small cohorts, observational designs, heterogeneous biospecimen procedures, and limited independent replication further restrict reproducibility and generalizability [18,73,80].

#### 4.5.3. Future Directions

Addressing these limitations will require:

1. Standardization and harmonization of microbiome-profiling methods, including sample handling, DNA extraction, sequencing, analytical workflows, and quality control [73,80].
2. Adequately powered, preferably prospective, multicenter studies involving diverse populations and standardized biospecimen collection to improve reproducibility and generalizability [18].
3. Independent external validation of proposed microbial biomarkers in cohorts distinct from those used for biomarker discovery, with prespecified analytical procedures and clinically relevant endpoints [69].
4. Careful measurement and control of biological and clinical confounders, including antibiotic and PPI exposure, treatment regimen, and previous anticancer therapy [18].
5. Validation across geographically and demographically diverse populations using harmonized analytical procedures to establish generalizability and clinical utility [18,73].
6. Prospective evaluation of clinical utility to determine whether microbiome-based biomarkers improve patient stratification or treatment-response prediction beyond existing clinical and molecular predictors [18].

## 5. Therapeutic Strategies: Restoring Symbiosis

### 5.1. Fecal Microbiota Transplantation (FMT)

Much of the clinical evidence linking microbiome modulation to immune-checkpoint inhibitor efficacy has been generated in melanoma and other non-GI cancers. These studies are included as comparative evidence to illustrate mechanisms and translational principles that may inform microbiome-based strategies in GI malignancies; however, their clinical outcomes should not be assumed to be directly transferable to GI cancers, given differences in tumor biology, treatment context, and microbiome characteristics.

Two distinct clinical contexts should be distinguished when evaluating FMT in oncology. In patients with metastatic melanoma refractory to anti-PD-1 therapy, responder-derived FMT combined with anti-PD-1 reinduction produced objective tumor responses in 3 of 10 patients in one phase 1 study, whereas a second study reported clinical benefit in 6 of 15 patients, including objective responses and durable disease control [57,58]. These findings provide preliminary proof-of-concept that FMT may modulate antitumor responses to anti-PD-1 therapy, but the studies were small, single-arm, early-phase trials without randomized controls and therefore do not establish efficacy.

In a separate clinical context, FMT has been investigated for treatment-refractory ICI-induced colitis. In a 12-patient case series, all patients had grade 3–4 ICI-related diarrhea or colitis that had failed to respond to corticosteroids and second-line immunosuppression with infliximab or vedolizumab. Ten patients (83%) experienced symptom improvement after FMT, and 92% achieved clinical remission by the end of the study period [78]. These colitis-related outcomes are distinct from objective tumor response and should not be interpreted as evidence of antitumor efficacy. Thus, FMT for refractory ICI-induced colitis represents a distinct clinical application from its investigational use to overcome resistance to immunotherapy.

### 5.2. Probiotics and Dietary Interventions

#### 5.2.1. Probiotics and Live Bacterial Supplementation

Probiotics and other live bacterial preparations have been investigated to modify the gut microbiota and influence cancer treatment outcomes. Clinical studies have reported potential benefits, particularly for treatment-related GI toxicity, but evidence for direct effects on tumor response and survival remains limited and heterogeneous. Findings from preclinical studies should therefore not be interpreted as evidence of clinical efficacy.

One of the better-characterized clinical examples is *Clostridium butyricum* MIYAIRI 588 (CBM588), a live bacterial preparation evaluated in patients with metastatic renal cell carcinoma (RCC) receiving immune checkpoint blockade. In a small randomized, open-label phase 1 study, CBM588 combined with nivolumab plus ipilimumab was associated with longer PFS than nivolumab plus ipilimumab alone (12.7 vs. 2.5 months; HR 0.15, 95% CI 0.05–0.47; *p* = 0.001), while the objective response rate was 58% versus 20% (*p* = 0.06) [81]. However, the study’s primary microbiome endpoint, which assessed changes in relative *Bifidobacterium* abundance from baseline to 12 weeks, was not met, and no significant between-group differences were observed in *Bifidobacterium* abundance or Shannon diversity [81]. Thus, although the trial provides a clinical signal for CBM588, it did not establish that the observed clinical benefit was mediated by the measured microbiome changes.

Longer-term follow-up of the same randomized phase Ib study, reported in a 2025 American Society of Clinical Oncology (ASCO) analysis, showed a median PFS of 36.2 versus 2.5 months (HR 0.18, 95% CI 0.07–0.47; *p* = 0.001), while the objective response rate remained 58% versus 20% (*p* = 0.06) [82]. These findings remain exploratory because of the very small sample size and single-center, open-label design of the original study. Larger randomized studies with prespecified microbiome and clinical endpoints are needed to determine whether bacterial supplementation provides reproducible clinical benefit.

Accordingly, probiotics and live bacterial supplementation should currently be considered investigational microbiome-based strategies rather than established cancer therapies. Their effects may depend on bacterial strain, host microbiome, cancer type, concomitant treatment, and clinical context, emphasizing the need for strain-specific characterization and adequately controlled clinical evaluation.

#### 5.2.2. Dietary Modulation

Dietary patterns can shape gut microbial communities and metabolite production relevant to cancer biology. Higher-fiber dietary patterns have been associated with lower CRC risk, whereas Western-style diets can promote microbial and metabolic alterations, including increased production of secondary bile acids such as DCA, which can contribute to colorectal tumorigenesis [10].

Preclinical studies suggest that ketogenic diets and their principal ketone body, 3-hydroxybutyrate, may enhance the antitumor effects of immune checkpoint blockade through immunometabolic mechanisms. However, these findings are derived primarily from experimental tumor models and do not establish ketogenic diets as a clinical adjunct to immunotherapy [83].

Microbial metabolites can exert context-dependent effects on antitumor immunity and ICI responses. Higher circulating butyrate and propionate levels have been associated with poorer outcomes during anti-CTLA-4 therapy, whereas TMAO has been associated with enhanced antitumor immunity and better immunotherapy responses in triple-negative breast cancer [30,54]. These contrasting observations highlight the complexity of translating dietary or metabolite-directed interventions into clinical practice.

Overall, dietary interventions remain supported predominantly by observational, mechanistic, and preclinical evidence, although prospective dietary intervention trials are emerging. Controlled clinical evidence demonstrating that a specific microbiome-directed dietary intervention reproducibly improves cancer treatment efficacy or patient outcomes remains limited. Accordingly, dietary modulation should currently be regarded as an investigational or supportive strategy rather than an established microbiome-based cancer therapy.

### 5.3. Engineered Microbial Therapeutics

Engineered and functionalized probiotic bacteria represent an emerging preclinical strategy in which bacterial platforms can be designed to preferentially target tumor tissues, modulate the tumor-associated microbiota and immune microenvironment, and deliver functional payloads to enhance antitumor responses [76,84].

‑ LGG@Ga-poly: *Lactobacillus rhamnosus* GG functionalized with a gallium–polyphenol network targeted pancreatic tumors, modulated the intratumoral microbiota, reduced immunosuppressive myeloid populations, increased tumor-infiltrating cytotoxic CD8^+^ T cells, and enhanced ICB in preclinical pancreatic cancer models [76].
‑ Engineered probiotic consortium: An engineered probiotic consortium using an orthogonal quorum-sensing system integrated tumor-microenvironment signals, including pH, hypoxia, and lactate, to coordinate release of therapeutic payloads including LdhA and an anti-PD-L1 nanobody. In humanized mouse models of CRC, this approach suppressed tumor progression and enhanced antitumor T-cell responses [85].
‑ ProCAR platform: The ProCAR platform uses engineered probiotics to release synthetic CAR targets within tumors, effectively labeling tumor tissue for recognition by engineered chimeric antigen receptor T cells and enabling antigen-agnostic targeting of otherwise difficult-to-target solid tumors in preclinical models [86].

Clinical Status:

Clinical translation of engineered bacterial therapeutics remains at an early stage. The attenuated *Salmonella Typhimurium* strain VNP20009 (VNP20009) was evaluated in a phase I study involving 24 patients with metastatic melanoma and 1 patient with metastatic RCC. Focal tumor colonization and dose-related increases in proinflammatory cytokines were observed, but no objective tumor regression was demonstrated, including in patients with tumor colonization [87].

Saltikva (*Salmonella*-IL2), an attenuated *Salmonella Typhimurium* strain engineered to express human IL-2, has also been evaluated clinically in a non-randomized phase II study in combination with FOLFIRINOX for metastatic pancreatic cancer [88]. In the final publication, among the 20 patients who received more than five doses of *Salmonella*-IL2 plus FOLFIRINOX, median overall survival was 20.3 months, median PFS was 15 months, and the objective response rate was 70% [88]. These outcomes should not be interpreted as estimates for the entire enrolled cohort because the >5-dose analysis represents a selected subgroup and included expanded-access patients. The study was non-randomized and relied on historical rather than concurrent controls, limiting causal comparisons with standard FOLFIRINOX. Conditioning the analysis on receipt of more than five doses may also introduce selection and guarantee-time bias, while historical comparisons are susceptible to differences in patient characteristics, treatment era, and clinical management. Thus, the findings provide early clinical evidence and a hypothesis-generating efficacy signal but do not establish a comparative survival benefit over standard FOLFIRINOX.

The principal challenges for engineered microbial therapeutics include safety, controllability, stability, heterogeneous tumor colonization, and translation from preclinical models to clinical practice. Engineered bacteria must achieve adequate tumor localization and therapeutic activity while maintaining control over bacterial behavior, payload expression, and in vivo stability [76,85]. Tumor localization and therapeutic activity may also vary according to bacterial strain, engineering properties, tumor characteristics, and route of administration [76]. Most platforms remain preclinical, and important translational questions include biodistribution, persistence, manufacturing, safety, and reproducibility of therapeutic effects in clinical settings [76,84,85].

Table 7 encompasses distinct microbial compartments, intervention types, and clinical endpoints. FMT and bacterial supplementation primarily target the intestinal microbial ecosystem, whereas engineered bacterial platforms such as VNP20009 and *Salmonella*-IL2 use bacteria as therapeutic delivery or immune-modulating agents rather than as interventions intended to restore intestinal symbiosis [78,87,88]. Clinical endpoints also differ: objective response rate is an antitumor efficacy endpoint, whereas symptom response or clinical remission may refer to treatment-related toxicity or inflammatory complications such as ICI-induced colitis [78,87]. Accordingly, these studies should be interpreted according to their respective biological targets and outcome measures rather than as directly interchangeable forms of clinical evidence.

## 6. Managing Treatment-Related Toxicity: Chemotherapy, Radiotherapy, and Immunotherapy

Cancer treatment-related toxicity can lead to treatment interruption, dose reduction, hospitalization, and impaired quality of life. The gut microbiome may modify treatment-related toxicity through microbial metabolism, intestinal barrier integrity, microbial metabolites, and host immune responses, but the evidence and clinical maturity differ substantially among chemotherapy, radiotherapy, and immunotherapy [61,67,84]. This section therefore distinguishes mechanistic, observational, and interventional evidence for microbiome-associated toxicity and its potential mitigation.

### 6.1. Chemotherapy-Related Toxicity

Chemotherapy commonly causes GI toxicity, including diarrhea, mucositis, nausea, vomiting, and disruption of intestinal barrier function [60]. Reported frequencies vary according to chemotherapy regimen and dose intensity, with substantially higher rates reported in some high-dose settings [60]. The gut microbiota may contribute to chemotherapy-associated toxicity through microbial drug metabolism, production of bioactive metabolites, disruption of intestinal barrier integrity, and modulation of host inflammatory pathways [60,64].

#### 6.1.1. Microbial Mechanisms of Chemotherapy Toxicity

Bacterial drug metabolism and toxicity. Enrichment of the bacterial preTA operon during oral fluoropyrimidine treatment has been associated with reduced fluoropyrimidine toxicity, while experimental studies demonstrate that preTA-expressing bacteria can inactivate 5-FU and ameliorate treatment-related toxicity [16]. Conversely, the microbiota-derived metabolite 2-methylisocitrate can enhance 5-FU activity through inhibition of mitochondrial isocitrate dehydrogenase, disruption of nucleotide homeostasis, and induction of DNA damage [15]. Together, these findings demonstrate that microbial metabolism can influence both anticancer drug activity and treatment-associated toxicity [15,16].

Bacterial β-glucuronidases and irinotecan toxicity. Bacterial β-glucuronidases can deconjugate the inactive irinotecan metabolite SN-38G to the cytotoxic metabolite SN-38 in the intestinal lumen, contributing to irinotecan-associated GI toxicity [64]. Pharmacological inhibition of bacterial β-glucuronidase activity has been investigated as a strategy to reduce irinotecan-induced intestinal toxicity, with preclinical studies demonstrating reduced diarrhea without compromising antitumor efficacy [91]. This approach remains experimental and requires clinical evaluation.

Cisplatin-associated toxicity. Preclinical evidence suggests that selected probiotic strains, including *L. reuteri* and *Clostridium butyricum*, may attenuate cisplatin-induced renal injury through modulation of gut microbiota composition, increased butyrate production, and suppression of renal inflammatory and oxidative responses [63]. These findings remain preclinical and strain-specific and should not be interpreted as evidence that probiotic supplementation prevents cisplatin toxicity in patients.

#### 6.1.2. Probiotic Mitigation Strategies

Microbiome-directed interventions have also been investigated for the prevention or attenuation of chemotherapy-associated toxicity. In a randomized, single-blind, placebo-controlled study of 100 patients with CRC receiving postoperative chemotherapy, a probiotic formulation containing *B. infantis*, *L. acidophilus*, *E. faecalis*, and *B. cereus* reduced chemotherapy-associated GI complications and partially restored chemotherapy-associated alterations in gut microbial composition. The intervention also increased fecal concentrations of SCFAs, including acetate, butyrate, and propionate [61]. These findings provide preliminary clinical evidence for a potential protective effect of this specific formulation but should not be generalized to other probiotic preparations or cancer populations.

A separate preclinical study in a genetically engineered mouse model of pancreatic ductal adenocarcinoma reported that combining *Lactobacillus paracasei* GMNL-133 and *Lactobacillus reuteri* GMNL-89 with gemcitabine reduced the severity of pancreatic intraepithelial neoplasia and expression of Ki-67 and vimentin. The combination was also associated with lower serum alanine aminotransferase and aspartate aminotransferase levels [62]. These findings remain preclinical and do not establish clinical efficacy.

Overall, mechanistic evidence for microbiome-mediated chemotherapy toxicity remains predominantly preclinical, with limited clinical evidence from specific probiotic formulations [61,64]. Accordingly, microbiome-based mitigation of chemotherapy toxicity should currently be regarded as preliminary and investigational rather than established supportive therapy.

### 6.2. Radiotherapy-Related Toxicity

Radiotherapy involving the abdomen or pelvis can cause acute and chronic GI toxicity, including radiation enteritis, diarrhea, abdominal pain, and persistent bowel dysfunction [67]. The gut microbiome is increasingly being investigated as a potential modifier of radiation-associated tissue injury and treatment response [65,66,67].

Radiation-associated intestinal injury may be accompanied by alterations in intestinal microbial composition and metabolic activity. These changes may influence local inflammation, microbial metabolite production, and host immune responses and may contribute to radiation-associated GI toxicity [66,67]. In a prospective observational cohort of patients with locally advanced cervical cancer receiving concurrent chemoradiotherapy, intestinal microbial alterations were associated with the severity and duration of radiation-enteritis-related diarrhea, supporting a relationship between microbiome characteristics and treatment toxicity without establishing causality [67].

#### Preclinical Evidence for Microbiome-Mediated Modulation

In preclinical models of microsatellite-stable CRC, antibiotic-mediated disruption of the gut microbiota reduced the efficacy of combined radiotherapy and immunotherapy, whereas administration of *B. fragilis* or the microbial metabolite propionate enhanced CD8^+^ T-cell infiltration and treatment responses [68]. These findings provide experimental evidence that the gut microbiota can influence antitumor immune responses during combined radiotherapy and immunotherapy. However, because these findings derive from preclinical models, they do not establish the efficacy or safety of microbiome-directed interventions for radiation-associated toxicity in patients.

Overall, human evidence linking the microbiome to radiotherapy toxicity remains predominantly observational, whereas mechanistic evidence is more developed in experimental models [65,66,67]. Microbiome-based approaches to radiotherapy toxicity therefore remain investigational rather than established supportive therapies.

### 6.3. Immune Checkpoint Inhibitor–Related Adverse Events

The gut microbiota has been associated with both the efficacy and toxicity of ICI therapy. Microbial diversity, specific bacterial taxa, and microbial metabolites have been investigated as potential modifiers of ICI efficacy, while associations between gut microbial profiles and irAEs have also been reported [50,59,92]. Microbial metabolites, including SCFAs and bile-acid derivatives, may influence intestinal immune regulation and systemic antitumor immunity [30,84]. However, many reported relationships remain observational and may be influenced by treatment regimen, antibiotic exposure, disease characteristics, diet, and other host factors [50,84].

#### 6.3.1. Microbiome–Colitis Associations

Alterations in gut microbial composition have been associated with ICI-associated colitis, and microbiome-directed interventions such as FMT have been investigated in patients with refractory ICI-induced colitis [78]. In melanoma, microbial configurations enriched in *Faecalibacterium* and other Firmicutes have been associated with favorable responses to anti-CTLA-4 therapy, although the association between *Faecalibacterium* abundance and colitis was not statistically significant in the cited cohort [30]. These observations illustrate that microbial features associated with improved antitumor immunity may overlap with features associated with immune-mediated toxicity and therefore cannot automatically be interpreted as protective against irAEs.

#### 6.3.2. FMT for Refractory ICI-Induced Colitis

FMT has been investigated as a microbiome-restoring intervention for treatment-refractory ICI-induced colitis. In a case series of 12 patients with grade 3–4 ICI-related diarrhea or colitis refractory to corticosteroids and second-line immunosuppression, 10 patients (83%) experienced symptom improvement after FMT, and 92% achieved clinical remission by the end of the study period [78]. Among patients achieving complete clinical responses, FMT was associated with increased microbial α-diversity and increases in specific bacterial taxa, including *Collinsella* and *Bifidobacterium* [78].

These findings provide preliminary clinical evidence for FMT in refractory ICI-induced colitis. Importantly, the reported endpoints were toxicity-related clinical outcomes, including symptom improvement and clinical remission, rather than antitumor objective response, and the study involved only 12 patients without a control group [78]. FMT for ICI-induced colitis should therefore currently be regarded as an investigational intervention requiring confirmation in larger, prospective controlled studies.

### 6.4. Microbiome-Based Mitigation Strategies

Microbiome modulation—including FMT, probiotics, dietary interventions, and other microbiome-directed approaches—is being investigated with the aim of reducing treatment-related toxicity while preserving or improving antitumor activity [84]. The clinical maturity of these approaches differs substantially, ranging from preclinical and observational studies to early clinical investigations [93].

#### 6.4.1. Probiotic and Other Microbiome-Directed Interventions

Selected probiotics and live bacterial preparations are being investigated for their potential to modulate intestinal inflammation and microbiome-associated immune functions during cancer treatment. Higher abundance of *Faecalibacterium* and related Firmicutes has been associated with favorable outcomes in some ICI-treated cohorts, while experimental studies indicate that specific commensal bacteria and microbial metabolites can modulate regulatory immune pathways and intestinal inflammation [30,94]. These observations do not establish probiotic supplementation as an effective strategy for preventing irAEs or improving cancer treatment outcomes.

#### 6.4.2. Dietary Factors

Dietary composition can influence gut microbial composition and metabolic activity, including the production of SCFAs and bile-acid derivatives, which may affect host immune responses and cancer treatment outcomes [84]. Experimental evidence further suggests that diet-related microbial metabolites, such as TMAO, can influence antitumor immunity and response to immunotherapy [54]. However, clinical evidence that a specific dietary intervention reliably prevents irAEs or other treatment-related toxicities remains limited. Dietary interventions should therefore currently be considered investigational or supportive approaches rather than established microbiome-directed cancer therapies.

#### 6.4.3. Antibiotic Stewardship

Antibiotic exposure around immune checkpoint inhibitor (ICI) treatment has been associated with poorer progression-free and/or overall survival in patients with melanoma, non-small-cell lung cancer (NSCLC), and renal carcinoma, although the strength of these associations varies by tumor type and study [95]. In older adults with lung cancer receiving PD-1 inhibitors, antibiotic use within 60 days before or after treatment initiation was associated with shorter overall survival (HR = 1.273, 95% CI: 1.104–1.469, *p* = 0.0009) [52]. These findings may partly reflect treatment-associated microbiome disruption, including reduced microbial diversity and altered community composition, which could influence antitumor immune signaling. Microbial metabolites may also modulate immune responses during checkpoint blockade [30,96].

However, these observational associations do not establish that antibiotics directly impair ICI efficacy or cause immune-related adverse events (irAEs). Antibiotic exposure may instead reflect infection, hospitalization, comorbidity, disease severity, or other factors independently associated with cancer outcomes. Moreover, reported associations between antibiotic exposure and irAEs remain inconsistent [52,95,97,98]. Antibiotics should therefore be prescribed according to clear clinical indications and antimicrobial-stewardship principles, with appropriate agent selection and treatment duration and avoidance of unnecessary broad-spectrum exposure where clinically appropriate [84,97]. The effects of antibiotic exposure on other microbiome-dependent outcomes, including chimeric antigen receptor T-cell (CAR-T) responses, also warrant further investigation [84]. Whether microbiome-preserving antibiotic strategies can improve cancer outcomes or reduce treatment-related toxicity requires prospective evaluation.

### 6.5. Risk Stratification and Monitoring

Microbial signatures have been associated with treatment outcomes and irAEs in ICI-treated patients and are being investigated as potential biomarkers for patient stratification [50]. Longitudinal microbiome profiling may help characterize treatment-associated microbial changes and identify candidate features associated with treatment-related toxicity, but such approaches remain investigational [97]. Their reproducibility and clinical utility remain uncertain, and prospective validation in standardized patient populations is required.

Antibiotic exposure should be incorporated into such models as a potentially relevant clinical variable rather than treated as a standalone causal biomarker. Future studies should integrate baseline and longitudinal microbiome measurements with antibiotic exposure, diet, treatment regimen, disease characteristics, and other clinically relevant covariates. Such multidimensional approaches may improve the reproducibility and potential clinical utility of microbiome-based toxicity prediction, but this requires prospective validation in well-characterized patient populations [88,98].

### 6.6. Cross-Modality Comparison of Microbiome–Toxicity Interactions

The evidence summarized above differs substantially among chemotherapy-, radiotherapy-, and ICI-associated toxicity. Mechanistic evidence supports microbiome involvement across all three treatment settings, but the availability and maturity of human clinical and interventional evidence differ considerably [93,99]. The reported interactions between the microbiome and treatment-related toxicities across different cancer treatment modalities are summarized in Table 8.

## 7. Limitations and Research Priorities: An Integrated Framework

The gut microbiota interacts dynamically with the host through metabolic, immune, and ecological processes [100]. Increasing evidence links microbial composition and function with cancer biology, including CRC progression, tumor immunity, and treatment response [20,92,101]. However, these relationships are heterogeneous and may vary according to tumor type, host characteristics, diet, treatment exposure, and other environmental factors [84,92]. Consequently, the clinical utility and causal significance of many microbiome-associated findings remain incompletely established [74,84].

Microbial effects should therefore be interpreted in their biological and clinical context rather than classified simply as harmful or beneficial. Their consequences may depend on microbial identity and functional properties, community context, host characteristics, tumor type, and treatment setting [30,84,92]. These considerations provide the basis for the integrated limitations and research priorities summarized below.

### 7.1. Integrated Limitations and Research Priorities

The major limitations identified across the current evidence base, together with corresponding priorities for methodological standardization, validation, and clinical translation, are summarized in Table 9.

### 7.2. Evidence Maturity and Clinical Translation

The evidence reviewed in this article can be considered at three broad levels of maturity: mechanistically supported, clinically promising, and insufficiently validated. This author-defined framework is intended to distinguish the strength and translational maturity of the available evidence rather than to imply equivalent certainty within each category [7].

#### 7.2.1. Mechanistically Supported Findings

Several microbiome–cancer interactions are supported by convergent experimental and translational evidence. These include bacterial genotoxin-associated epithelial injury and genomic damage, chronic inflammation, microbial metabolite-mediated modulation of host cellular and immune functions, and *F. nucleatum*-associated chemoresistance through mechanisms involving TLR4/MYD88-dependent autophagy, ferroptosis inhibition, and suppression of chemotherapy-induced pyroptosis [12,24,30,31,32,46,78]. Nevertheless, the relevance of these mechanisms to human tumors may depend on bacterial strain, tissue compartment, microbial community context, host factors, and treatment setting.

*F. nucleatum* illustrates both the promise and the complexity of microbiome-based cancer research. Evidence from experimental and clinical studies links its enrichment with CRC development, immune modulation, and treatment response, but the strength and clinical applicability of these associations vary according to the microbiome compartment, disease stage, treatment context, and analytical method. Its repeated identification across mechanistic and translational studies supports further investigation, although prospective studies are needed to determine whether *F. nucleatum* can serve as a reliable biomarker or therapeutic target. Standardized sampling, longitudinal assessment, and integration with host and tumor characteristics will be important for establishing its clinical utility.

#### 7.2.2. Clinically Promising Findings

Clinical studies have reported associations between gut microbial diversity or composition and ICI outcomes, as well as associations between antibiotic exposure around ICI treatment and clinical outcomes in several tumor types [14,19,50,74]. Early clinical studies of FMT have reported objective tumor responses in subsets of patients with anti-PD-1-refractory melanoma, whereas separate studies have reported clinical improvement and remission in patients with treatment-refractory ICI-induced colitis [57,58,78]. These findings provide clinical proof-of-concept but remain insufficient to establish routine microbiome-directed treatment strategies.

#### 7.2.3. Insufficiently Validated Findings

Several microbiome-directed approaches remain investigational, including therapeutic probiotics and other live microbial products, engineered tumor-targeting bacteria, and many microbiome-based biomarkers outside selected CRC screening applications [23,81,85,86,90]. Although some strategies have generated encouraging mechanistic or early clinical findings, their evidence maturity, clinical applicability, and safety profiles remain heterogeneous. Clinical translation will require adequately powered controlled studies, independent validation, standardized endpoints, and longer-term safety assessment.

The translational potential and evidence maturity of the microbiome-based cancer strategies discussed in this section are summarized in Table 10.

### 7.3. Engineered Microbial Therapeutics in Context

Engineered tumor-targeting bacteria, including platforms such as VNP20009 and Saltikva, represent a distinct therapeutic paradigm from interventions designed primarily to restore or reshape the intestinal microbiome. These approaches exploit microbial properties for tumor localization, local immune modulation, and delivery of therapeutic payloads [76,85,86,87].

Most engineered bacterial cancer therapeutics remain in preclinical or early clinical development. Recent studies provide proof-of-concept for tumor targeting and therapeutic delivery, but the available evidence remains limited by preclinical study designs and, where clinical studies have been conducted, small or early-phase cohorts, non-randomized or open-label designs, and limited follow-up [76,85,86,90]. Engineered microbial therapeutics should therefore be regarded as an emerging component of the microbiome–cancer field rather than an established treatment modality.

## 8. Conclusions

The gut microbiome is an important component of cancer biology, with evidence linking microbial communities and their functions to carcinogenesis, antitumor immunity, treatment response, and treatment-related toxicity. Mechanistically supported interactions include bacterial genotoxin-associated genomic damage, inflammation, microbial metabolite-mediated host and immune modulation, and *F. nucleatum*-associated chemoresistance [12,24,31,46,77]. Clinical studies further suggest that the microbiome can influence immunotherapy outcomes and treatment-related toxicity, while early FMT and other microbiome-directed interventions provide clinical proof-of-concept in selected settings [14,19,57,58,78].

Taken together, the field has progressed from descriptive associations toward increasingly defined mechanisms and early clinical translation, but the evidence remains heterogeneous and context-dependent. The central challenge is now to determine which microbiome-associated findings are reproducible and clinically actionable across patients, tumor types, treatments, and biological compartments. Microbiome-directed strategies therefore represent a promising component of cancer research, but their incorporation into routine prevention, treatment, or supportive care should be guided by validated biomarkers, reproducible clinical benefit, and an appropriate evidence base.

## Figures and Tables

**Table 1:** Microbial Sampling Compartments in Cancer Research: Definitions, Methods, and Interpretive Caveats.

Compartment	Definition	Typical Sampling and Analytical Approaches	Ecological and Immunological Context	Representative Cancer Associations	Key Interpretive Caveat
Faecal	Microbes recovered from stool, representing mainly luminal intestinal communities	Stool; 16S rRNA sequencing, shotgun metagenomics, metabolomics	Reflects luminal ecology and is influenced by diet, transit, and host physiology	CRC screening and risk stratification; FMT; ICI response	Does not fully represent mucosa-associated or tumor-associated communities
Mucosa-associated	Microbes associated with the intestinal mucus layer or epithelium	Endoscopic biopsy, mucosal sampling or lavage; 16S rRNA sequencing, metagenomics, spatial analyses	Reflects local epithelial interactions and mucosal immunity	CRC and other GI malignancies; inflammatory and treatment-related mechanisms	Varies by anatomical site, sampling depth, and inflammatory status
Oral	Microbes of the oral cavity, including saliva, tongue, and dental plaque	Saliva, oral swab, tongue scraping, or plaque; sequencing and microbial profiling	Influenced by oral health, hygiene, diet, and host immunity	CRC and other GI cancers; *F. nucleatum*-related findings	Oral profiles should not be assumed to represent gut communities
Intratumoral	Microbes detected within tumor tissue, including intracellular and extracellular niches	Tumor resection or biopsy; 16S rRNA sequencing, metagenomics, spatial or in situ methods	Shaped by the tumor microenvironment, immunity, oxygenation, and local metabolism	CRC, gastric, pancreatic, breast, lung, and other cancers	Low biomass increases contamination risk; findings are tissue- and cancer-specific
Tumor-adjacent/normal tissue	Microbes detected in tissue adjacent to the tumor or histologically normal tissue from the same patient	Surgical margins or adjacent-tissue biopsies	Provides a within-patient comparator and may reflect local or field effects	Comparative studies across GI and other solid tumors	Histologically normal tissue is not necessarily equivalent to healthy tissue

Notes to Table 1. Microbial compartments are not interchangeable; findings should be interpreted according to the anatomical source and should not be extrapolated across compartments without appropriate validation. The pancreatic gut-microbiota nomogram discussed in this review is classified according to its actual sampling source and should not be assumed to represent an intratumoral microbiome model. Engineered tumor-targeting bacteria are therapeutic agents rather than endogenous microbiome-modulating interventions and are therefore considered separately. Low-biomass tissue compartments require stringent contamination controls and appropriate negative controls.

**Table 2:** Microbial Metabolites Implicated in GI Carcinogenesis.

Metabolite Class	Metabolite(s)	Source	Key Mechanism(s)	Effect
SCFAs	Butyrate, propionate	Microbial fermentation of dietary fiber	Histone lysine acylation, chromatin regulation, gene-expression modulation; butyrate can also inhibit histone deacetylases (HDACs)	Increased secondary-bile-acid production and DCA can promote CRC development [10]
Bile acids	DCA	Microbial transformation of primary bile acids, particularly through bacterial 7α-dehydroxylation	Promotes colonic epithelial proliferation and tumor-promoting signaling	Potentially tumor-promoting; increased bacterial secondary-bile-acid production and DCA can promote CRC development [10]
Tryptophan metabolites	Indole derivatives, including I3A	Microbial tryptophan metabolism	AhR activation and modulation of immune-cell function	Context-dependent; specific microbial indole metabolites can enhance antitumor CD8^+^ T-cell responses [29]

**Table 3:** Integrated Mechanistic Evidence Linking the Gut Microbiome to GI Carcinogenesis.

Mechanism	Evidence Level	Key Strengths	Key Limitations	Key References
Pro-inflammatory signaling	Preclinical + translational	Multiple inflammatory pathways; supporting human tissue evidence	Context dependence; confounding by diet and host factors	[11,12,22]
Metabolic dysregulation	Preclinical + translational	Direct metabolite–host interactions; links with diet and epithelial biology	Concentration dependence; local versus circulating effects may differ	[5,10,36]
Genotoxicity	Preclinical + human mutational signatures	Direct DNA-damage mechanisms; characteristic mutational patterns	Strain variability; causal in vivo relevance remains incompletely established	[12,31,32]
Epigenetic modifications	Predominantly preclinical	Evidence for microbial- and metabolite-associated epigenetic effects	Few causal human in vivo studies	[33,35,36]
Immune suppression and evasion	Preclinical + translational	Multiple mechanisms involving TIGIT, PD-1/PD-L1, MDSCs, and inflammatory immune pathways	Strong context dependence; effects may differ across tumor types and tissue compartments	[22,25,27,38]

**Table 4:** Key Cancer-Associated Bacteria in GI Cancers.

Bacterium	Principal Mechanisms	Cancer Types	Key References
*F. nucleatum*	Fap2–TIGIT interaction; inflammatory CAF remodeling; chemoresistance; tumor-associated neutrophil recruitment	CRC, gastric cancer	[20,22,24,38]
ETBF	BFT-mediated signaling; NF-κB/STAT3-related responses; IL-17 responses; MDSC recruitment	CRC	[11,12,28]
pks^+^*E. coli*	Colibactin production; DNA damage; genotoxic stress	CRC	[12,31]
*H. pylori*	CagA/VacA-associated virulence; chronic inflammation; CagA-dependent PD-L1 upregulation; M2-like macrophage polarization	Gastric cancer	[39,41,45,46]
*Peptostreptococcus anaerobius*	Integrin α2β1/NF-κB/CXCL1 signaling; MDSC recruitment	CRC	[27]
*Shigella flexneri*	IL-1β/IL-6 induction; DNA damage; ERBB3/PI3K-AKT signaling	CRC	[47]

Abbreviations: CRC, colorectal cancer; ETBF, enterotoxigenic *Bacteroides fragilis*; MDSC, myeloid-derived suppressor cell; CAF, cancer-associated fibroblast; ERBB3, Erb-b2 receptor tyrosine kinase 3.

**Table 5:** Key Microbial Metabolites and Their Effects on Antitumor Immunity.

Metabolite Class	Metabolite	Source/Microbial Origin	Key Mechanism	Context-Dependent Effects	Key References
SCFAs	Butyrate, Propionate	Fermentation of dietary fiber by gut microbes	HDAC inhibition; histone acylation; metabolic and epigenetic regulation	Context-dependent: butyrate can enhance antitumor CD8^+^ T-cell function through ID2/IL-12 signaling and improve oxaliplatin efficacy, whereas elevated systemic SCFAs have been associated with reduced anti-CTLA-4 efficacy	[5,30,36]
Tryptophan metabolites	I3A	*Lactobacillus reuteri*; microbial tryptophan metabolism	AhR-dependent signaling and enhancement of CD8^+^ Tc1 effector function	Beneficial in preclinical models: enhances antitumor CD8^+^ T-cell immunity and ICI efficacy in preclinical melanoma models	[29]
Purine nucleosides	Inosine	Produced by gut bacteria, particularly *Bifidobacterium pseudolongum*	A_2_A receptor-dependent costimulation of antitumor T-cell responses; requires appropriate costimulation	Beneficial but context-dependent: enhances antitumor immunity and ICI efficacy in preclinical models; effects depend on A_2_A receptor signaling and the immune costimulatory environment	[55]
Bile Acids	DCA	Microbial transformation of primary bile acids	Suppresses CD8^+^ T-cell effector function via PMCA/Ca^2+^–NFAT2 inhibition	Detrimental: DCA suppresses antitumor CD8^+^ T-cell activity; bacteria harboring DCA-biosynthetic pathways can promote tumor growth	[53]
TMAO	TMAO	Host–microbial metabolism of choline and L-carnitine	Promotes CD8^+^ T-cell-mediated antitumor immunity and tumor-cell pyroptosis	Potentially beneficial: enhances immunotherapy efficacy in TNBC preclinical models; clinical evidence remains limited	[54]

**Table 6:** Performance Characteristics of Microbiome-Based Biomarkers in Cancer.

Biomarker/Model	Cancer	Sample Type	Study Population/Sample Size	Sensitivity	Specificity	AUC (95% CI)	Validation Design	Intended Use/Design	Reference
*F. nucleatum* + FIT	CRC	Stool/fecal	Primary cohort: *n* = 309; independent validation cohort: *n* = 181	92.3%	93.0%	0.95 (0.92–0.98)	Independent validation cohort; validation AUC 0.96 (0.92–0.99)	Screening-oriented CRC detection	[23]
*F. nucleatum* + FIT	AA	Stool/fecal	Primary cohort: *n* = 309; independent validation cohort: *n* = 181	38.6%	89.0%	0.65 (0.58–0.73)	Independent validation cohort; validation AUC 0.63 (0.55–0.72)	Detection of AA	[23]
Four-marker panel (*F. nucleatum*, *Lachnoclostridium* sp. m3, *C. hathewayi*, *B. clarus*) + FIT	CRC	Stool/fecal	*n* = 676; 210 CRC, 115 AA, 86 non-AA, 265 controls; 435 asymptomatic	90.9%	NR	NR	Previously established microbial score evaluated in symptomatic and asymptomatic cohorts	Non-invasive CRC screening	[71]
Four-marker panel + FIT	AA	Stool/fecal	*n* = 676; 115 AA; 435 asymptomatic participants	48.5%	NR	NR	Previously established microbial score evaluated in symptomatic and asymptomatic cohorts	Non-invasive detection of AA	[71]
*F. nucleatum* + *P. anaerobius* + methylated ITGA4 + FIT (pd-score)	CRC	Stool/fecal + FIT	Development cohort; independent external validation cohort *n* = 117; additional CRC screening cohort	95.38%	91.37%	0.958 *	Independent external validation; additional screening-cohort evaluation	Non-invasive detection of CRC and AA	[69]
*F. nucleatum* + *P. anaerobius* + methylated ITGA4 + FIT (pd-score)	AA	Stool/fecal + FIT	Development cohort; independent external validation cohort *n* = 117; additional CRC screening cohort	70.83%	91.37%	0.958 *	Independent external validation; AA sensitivity 80.00% in external validation cohort	Non-invasive detection of AA	[69]
*Salivary P. gingivalis* + *A. actinomycetemcomitans* + circulating miR-21 + miR-155 + diabetes	Pancreatic cancer	Saliva + blood	41 pancreatic cancer cases + 40 matched controls	87.8%	78.9%	0.878 (0.802–0.955)	No independent external validation reported	Non-invasive diagnostic/early-detection candidate	[70]
18-species gut bacterial model	ICI-treated HCC	Stool/fecal	*n* = 80; training *n* = 49, internal test *n* = 31	NR	NR	0.7563 (95% CI, 0.5834–0.9293)	Fivefold cross-validation in training cohort plus separate internal test cohort	Prediction of ICI treatment response/clinical benefit	[72]

* The AUC of 0.958 represents discrimination of the combined AA and CRC group from healthy controls, rather than an AUC specific to AA or CRC alone. Abbreviations: AA, advanced adenoma; AUC, area under the receiver operating characteristic curve; CI, confidence interval; CRC, colorectal cancer; FIT, fecal immunochemical test; HCC, hepatocellular carcinoma; ICI, immune-checkpoint inhibitor; NR, not reported. Note: Performance estimates from case–control or case-enriched studies should not be interpreted as equivalent to performance in intended population-screening settings. “Independent external validation” refers to evaluation in a cohort independent of the biomarker-development population rather than a randomly separated subset of the same development population. Sensitivity and specificity should be interpreted together with the study population, endpoint definition, assay threshold, and intended clinical use. For FIT-based combinations, increased sensitivity should be considered alongside specificity and the potential for false-positive results and downstream colonoscopy demand. The HCC model is a treatment-response prediction model rather than a diagnostic cancer-detection test.

**Table 7:** Clinical and Translational Evidence for Microbiome-Modulating and Microbial Therapeutic Strategies in Cancer.

Study/Intervention	Cancer Type/Condition	Design	Clinical Development Stage/Evidence Level	Sample Size	Intervention	Comparator	Primary Endpoint	Principal Outcome	AEs	Major Limitations
[57]	metastatic melanoma refractory to anti-PD-1 therapy	Phase I, single-arm	Early clinical; preliminary proof-of-concept	*n* = 10	Responder-derived FMT + anti-PD-1 reinduction	None	Safety and feasibility	Objective responses in 3/10 patients (1 CR, 2 PR)	No major FMT-related safety signal was established	Small sample; single-arm; non-randomized; highly selected anti-PD-1-refractory population
[58]	metastatic melanoma refractory to anti-PD-1 therapy	Clinical trial, single-arm	Early clinical; preliminary proof-of-concept	*n* = 15 evaluable	Responder-derived FMT + anti-PD-1 reinduction	None	Safety and clinical benefit	Objective responses in 3/15 patients (1 CR, 2 PR); clinical benefit in 6/15 patients, including stable disease in 3 patients	No major FMT-related safety signal was established	Small sample; non-randomized; selected anti-PD-1-refractory population
[89]	Advanced melanoma	Phase I, multicenter, single-arm	Early clinical; safety/feasibility with preliminary efficacy	*n* = 20	Healthy-donor oral FMT + anti-PD-1 therapy	None	Safety and feasibility	ORR 65% (13/20), including 4 complete responses; no separate overall clinical-benefit endpoint was reported	No grade 3 AEs were attributed to FMT alone; grade 3 immune-related AEs occurred in 25% during combination therapy; no grade 4/5 irAEs were reported	Small single-arm study; no randomized control; limited sample size; hypothesis-generating; melanoma population limits direct generalizability to GI malignancies
[78]	ICI-induced grade 3–4 diarrhea/colitis	Prospective case series	Early clinical; uncontrolled therapeutic evidence	*n* = 12	Healthy-donor FMT by colonoscopy	None	Clinical response and safety	Symptom improvement in 10/12 patients (83%); clinical remission in 11/12 (92%) by study end. These are colitis-specific endpoints and not antitumor response endpoints (ORR)	No FMT-related complications were reported within 7 or 30 days	Small uncontrolled case series; selected refractory population; no comparator; outcomes relate to ICI-induced colitis rather than tumor response
[81]	Metastatic RCC	Randomized, open-label phase Ib	Early clinical; randomized but exploratory evidence	*n* = 30 randomized; *n* = 29 in final efficacy analysis (19 CBM588/10 control)	CBM588 + nivolumab/ipilimumab	Nivolumab/ipilimumab alone	Prespecified microbiome endpoint: change in relative *Bifidobacterium* abundance at 12 weeks	Prespecified microbiome endpoint was not met; exploratory PFS was 12.7 vs. 2.5 months (HR 0.15, 95% CI 0.05–0.47; *p* = 0.001); ORR was 58% vs. 20% (*p* = 0.06)	No major between-group safety difference was reported	Very small, single-center, open-label study; clinical efficacy findings were exploratory; prespecified microbiome endpoint was negative
[82]	Metastatic RCC	Long-term follow-up of randomized phase Ib trial	Early clinical; exploratory follow-up	*n* = 29 (19 CBM588/10 control)	CBM588 + nivolumab/ipilimumab	Nivolumab/ipilimumab alone	Long-term clinical outcomes	Updated median PFS was 36.2 vs. 2.5 months (HR 0.18, 95% CI approximately 0.07–0.47; *p* = 0.001); ORR was 58% vs. 20% (*p* = 0.06)	No major between-group safety difference was highlighted in the updated report	Very small sample; single-center; updated efficacy remains exploratory and requires confirmation in larger randomized studies
[90]	Advanced/metastatic MSS CRC, TNBC, and checkpoint-inhibitor-relapsed solid tumors	Phase I/II, open-label, multicohort	Early clinical; limited preliminary clinical activity	*n* = 69	Oral EDP1503 + pembrolizumab	None	Safety/tolerability, objective response rate, and immune-response rate	Objective responses in 3/69 patients; clinical activity was limited. Among participants receiving 4 capsules twice daily, median PFS was 1.8 months and median OS was 7.8 months	Grade ≥ 3 treatment-emergent AEs occurred in 28/69 (40.6%); no new safety signals were identified	Early-phase; single-arm; heterogeneous population; limited clinical activity; requires controlled confirmation
[87]	Metastatic melanoma and metastatic RCC	Phase I, dose-escalation	Early clinical; feasibility/colonization evidence without demonstrated efficacy	*n* = 25 (24 melanoma, 1 RCC)	Intravenous attenuated *Salmonella Typhimurium* VNP20009	None	Safety, tumor colonization, and antitumor effects	Focal tumor colonization in 3 patients; no objective tumor regression	Dose-related toxicity, including thrombocytopenia, anemia, persistent bacteremia, hyperbilirubinemia, and GI toxicity	Small early-phase study; dose-related toxicity; no objective tumor regression
[88]	Stage IV metastatic pancreatic cancer	Phase II, non-randomized	Early clinical; preliminary feasibility/efficacy evidence	34 enrolled; 26 received combination therapy; 20 received >5 doses	Oral *Salmonella*-IL2 + FOLFIRINOX	Historical FOLFIRINOX outcomes	Overall survival, PFS, objective response, and safety	Among the selected subgroup receiving >5 doses, median OS was 20.3 months, median PFS was 15 months, and ORR was 70%	41 serious AEs were attributed to standard chemotherapy; none were attributed to *Salmonella*-IL2	Non-randomized; historical comparison; small sample; expanded-access patients included; >5-dose analysis represents a selected subgroup and may be subject to selection and guarantee-time bias; outcomes cannot be interpreted as estimates for the entire enrolled cohort
[76,85,86] *	Various preclinical cancer models	Preclinical translational studies	Preclinical; no established human clinical efficacy or safety	Model-dependent	Examples include functionalized *L. rhamnosus* GG, engineered probiotic consortia, and ProCAR platforms	Model-dependent	Tumor targeting, immune modulation, and antitumor activity	Enhanced tumor localization, immune activation, or tumor control in preclinical models	Model-dependent; human safety not established	Preclinical evidence only; animal-model findings cannot establish clinical efficacy or safety
[10,83]	Various cancers	Predominantly observational and preclinical studies	Observational/preclinical; clinical treatment efficacy not established	Model- and study-dependent	Dietary patterns, including high-fiber and ketogenic approaches	Study-dependent	Microbiome composition, metabolite production, cancer risk, or treatment response	Dietary patterns can alter microbial communities and metabolites; specific microbiome-directed dietary interventions have not established reproducible clinical treatment efficacy	Intervention- and study-dependent	Heterogeneous dietary exposures; substantial confounding in observational studies; limited controlled oncology trials; clinical benefit remains unvalidated

Abbreviations: AE, adverse event; CBM588, *Clostridium butyricum* MIYAIRI 588; CR, complete response; FMT, fecal microbiota transplantation; HR, hazard ratio; ICI, immune checkpoint inhibitor; irAE, immune-related adverse event; MSS, microsatellite stable; mPFS, median progression-free survival; mOS, median overall survival; ORR, objective response rate; PD-1, programmed cell death protein 1; PR, partial response; RCC, renal cell carcinoma; TNBC, triple-negative breast cancer. * Engineered microbial platforms are presented as a separate translational category because the principal examples discussed in this review remain preclinical and therefore should not be presented as human clinical trials.

**Table 8:** Microbiome–Toxicity Interactions Across Cancer Treatment Modalities.

Treatment Modality	Common Toxicities	Microbial Associations/Mechanisms	Strongest Current Evidence	Clinical Evidence	Microbiome-Based Mitigation	Clinical Readiness
Chemotherapy	Diarrhea, mucositis, nausea, vomiting, intestinal barrier disruption, and regimen-specific renal or hematologic toxicity	preTA operon; bacterial β-glucuronidases; 2-methylisocitrate; selected probiotic strains	Mechanistic and preclinical evidence for microbiome-mediated drug metabolism and treatment toxicity [15,16,64]	Randomized clinical trial showing reduced chemotherapy-associated GI complications with a specific probiotic formulation in CRC [61]	Probiotics, dietary approaches, and microbiome-directed drug-metabolism strategies	Preliminary; intervention- and regimen-specific
Radiotherapy	Radiation enteritis, diarrhea, abdominal pain, and other acute or chronic bowel dysfunction	Altered microbial composition and metabolites; *B. fragilis* and propionate in experimental models	Preclinical evidence linking microbial composition and metabolites to combined radiotherapy–immunotherapy response through CD8^+^ T-cell modulation [68]	Predominantly observational clinical evidence [65,66,67]	Dietary and microbiome-modulating approaches remain investigational	Investigational; predominantly observational/preclinical
Immunotherapy (ICI)	Colitis, diarrhea, hepatitis, endocrinopathies, and other irAEs	*Faecalibacterium*, Firmicutes, *Collinsella*; microbial metabolites and immune signaling	Translational and observational evidence linking microbiome features with ICI efficacy and toxicity [30,50,59]	Early clinical evidence supporting FMT for refractory ICI-induced colitis [78]	FMT, probiotics, dietary approaches, and antibiotic stewardship	Early clinical/investigational

Abbreviations: CRC, colorectal cancer; FMT, fecal microbiota transplantation; GI, gastrointestinal; ICI, immune checkpoint inhibitor; irAEs, immune-related adverse events.

**Table 9:** Integrated Limitations and Research Priorities in Microbiome-Based Cancer Research.

Limitation	Description	Corresponding Research Priority	Key References
Small clinical trials	Evidence remains substantially concentrated in small or early-phase studies, with limited reproducibility across cohorts and treatment settings	Conduct adequately powered randomized controlled trials with clearly defined efficacy and safety endpoints; incorporate longitudinal microbiome and metabolome profiling	[18,90]
Heterogeneity of methods	Differences in DNA extraction, sequencing, taxonomic profiling, and analytical approaches complicate cross-study comparisons	Harmonize microbiome-profiling workflows, including sample processing, DNA extraction, sequencing, and analytical procedures; establish biobanks of well-characterized donor stools and reference microbial consortia	[18,73,80]
Lack of standardized FMT protocols	Variation in donor selection, pathogen screening, stool preparation, administration, and dosing can complicate comparisons across studies; transmission of an ESBL-producing *E. coli* strain through FMT has caused bacteremia and death	Standardize FMT protocols for donor selection, pathogen screening, stool preparation, delivery route, dosing, and pretreatment; implement rigorous donor and pathogen screening	[78,102]
Population differences	Microbial signatures may not generalize across geographic, demographic, metabolic, and treatment contexts	Validate biomarkers and interventions across diverse and independent populations and establish their generalizability across relevant clinical contexts	[18,74]
Confounding factors	Medications, diet, previous treatments, and other clinical or host factors can confound microbiome–outcome associations	Carefully measure and control for biological and clinical confounders, including antibiotic and PPI exposure, treatment regimen, and previous anticancer therapy	[18,97]
Long-term safety	Long-term safety of sustained microbiome manipulation and administration of live microbial products remains incompletely characterized; FMT carries risks of transmission of pathogenic or antimicrobial-resistant organisms	Conduct long-term safety monitoring with extended follow-up; evaluate bacterial persistence, horizontal gene transfer, and unintended effects on the resident microbiome and host tissues	[97,102]
Lack of multi-omics integration	Taxonomic profiling alone may provide limited information about microbial functional activity and host–microbe interactions	Integrate metagenomics, metatranscriptomics, metabolomics, and host immune profiling with tumor genomic, transcriptomic, and immune profiling	[15,16,49]
Insufficient patient stratification	Many interventions are evaluated in heterogeneous populations, limiting detection of treatment-specific effects	Stratify patients according to baseline microbiome composition, host immune status, tumor type, treatment regimen, diet, medications, and other relevant host factors	[18,49,92]
Prognostic versus predictive confusion	Associations between microbial features and clinical outcomes do not necessarily demonstrate that a biomarker predicts differential benefit from a specific treatment	Distinguish prognostic from predictive biomarkers and validate predictive utility in randomized or appropriately stratified designs	[18,72,74]
Compartment non-interchangeability	Findings from fecal, mucosal, oral, and intratumoral compartments are often conflated	Use compartment-matched sampling and validate findings within the relevant biological compartment before extrapolation	[20,21,28]

Abbreviations: ESBL, extended-spectrum β-lactamase; FMT, fecal microbiota transplantation; PPI, proton pump inhibitor.

**Table 10:** Translational potential and evidence maturity of microbiome-based cancer strategies.

Microbiome-Based Strategy	Potential Clinical Application	Evidence Maturity	Representative Evidence	Key Limitations and Requirements for Translation
Microbiome profiling and biomarker development	Identification of microbial signatures associated with treatment response, resistance, prognosis, and treatment-related toxicity	Emerging/investigational	Longitudinal profiling of 175 patients with advanced melanoma receiving ICB identified microbiome features associated with progression-free and overall survival and showed that microbial dynamics varied according to treatment regimen, irAEs, and concomitant medications. In GI cancers, integrated gut and intratumoral microbiome analyses have also demonstrated associations with immunotherapy response [18,74].	Interindividual variability; differences among faecal, mucosa-associated, oral, and intratumoral compartments; methodological heterogeneity; effects of antibiotics, PPIs, diet, geography, and treatment; need for prospective and external validation
Probiotics and defined microbial consortia	Modulation of dysbiosis and treatment-related toxicity, with possible effects on anticancer treatment efficacy	Early clinical/investigational	Recent systematic reviews and meta-analyses indicate that probiotic or synbioticsupplementation can reduce selected chemotherapy-related complications and modify gut microbial composition, although evidence for effects on tumor-control outcomes remains less established [103,104].	Strain-specific effects, heterogeneous formulations and dosing, variable colonization, inconsistent clinical outcomes, and uncertain long-term safety
Faecal microbiota transplantation (FMT)	Restoration of microbial communities to overcome immunotherapy resistance or manage immune-checkpoint inhibitor-associated toxicity	Early clinical proof-of-concept	Clinical studies have shown that responder-derived or healthy-donor FMT can be combined with anti-PD-1 therapy in selected patients with advanced melanoma or other refractory cancers. FMT has also produced clinical improvement and remission in patients with refractory immune-checkpoint inhibitor-associated colitis [78,105].	Small cohorts, donor and recipient heterogeneity, protocol variability, variable engraftment, infectious-transmission risk, and limited long-term safety data
Microbial metabolite modulation	Therapeutic manipulation of microbial metabolites, including SCFAs, inosine, indole derivatives, bile acids, and TMAO, to influence tumor biology or antitumor immunity	Preclinical to early translational	Experimental studies indicate that microbial metabolites can modify immune-cell function, inflammation, epithelial biology, and therapeutic responses. TMAO, for example, has been shown experimentally to promote CD8^+^ T-cell-mediated antitumor immunity in triple-negative breast cancer [54,106].	Context-dependent biological effects, complex host–microbe metabolic interactions, uncertain dose–response relationships, and limited clinical validation
Engineered tumor-targeting bacteria	Selective tumor colonization and localized delivery of therapeutic molecules or modulation of the tumor microenvironment	Experimental to early clinical	Engineered bacteria can be programmed for tumor targeting, therapeutic payload delivery, and modulation of the tumor microenvironment. Recent analyses document expanding preclinical and clinical development of these platforms [107,108]. The specific VNP20009 phase I study demonstrated tumor colonization in 3 of 25 treated patients but no objective tumor regression [87].	Dose-related toxicity, bacteremia, limited tumor localization, host immune responses, tumor heterogeneity, manufacturing, and limited clinical efficacy
Microbiome-informed combination therapy	Integration of microbiome modulation with immune-checkpoint inhibitors, chemotherapy, or other systemic anticancer therapies	Emerging/investigational	Clinical and translational studies support investigation of microbiome modulation as an adjunct to ICB and other anticancer treatments. Recent work emphasizes microbiota-centered interventions, treatment–microbiome interactions, and the need for rational combination strategies [109,110,111].	Complex interactions among microbiota, drugs, antibiotics, PPIs, diet, host immunity, and tumor biology; optimal timing, sequencing, treatment combinations, and patient selection remain unresolved
Multi-omics and integrated patient stratification	Integration of microbiome, metabolomic, genomic, immune, and clinical data to identify biologically defined patient subgroups	Emerging/investigational	Longitudinal microbiome profiling and integrated gut–tumor analyses demonstrate the potential of microbiome-associated features for patient stratification. Recent multi-omics studies have further integrated microbial and host molecular data to identify clinically relevant cancer-associated patterns [3,18,74].	High-dimensional datasets, analytical reproducibility, standardization, interpretability, population heterogeneity, model overfitting, and the need for prospective external validation

Abbreviations: FMT, faecal microbiota transplantation; ICB, immune-checkpoint blockade; PD-1, programmed cell death protein 1; PPI, proton-pump inhibitor; SCFAs, short-chain fatty acids; TMAO, trimethylamine N-oxide.

## Data Availability

No new data were generated or analyzed for this narrative review. Data sharing is not applicable to this article.

## Abbreviations

The following abbreviations are used in this manuscript:

5-FU	5-fluorouracil
AA	Advanced adenoma
AE	Adverse event
AhR	Aryl hydrocarbon receptor
AUC	Area under the receiver operating characteristic curve
BFT	*Bacteroides fragilis* toxin
CAF	Cancer-associated fibroblast
CBM588	*Clostridium butyricum* MIYAIRI 588
CDT	Cytolethal distending toxin
CRC	Colorectal cancer
CTLA-4	Cytotoxic T-lymphocyte-associated protein 4
DCA	Deoxycholic acid
ETBF	Enterotoxigenic *Bacteroides fragilis*
FIT	Fecal immunochemical test
FMT	Fecal microbiota transplantation
GI	Gastrointestinal
ICB	Immune-checkpoint blockade
ICI	Immune-checkpoint inhibitor
irAE	Immune-related adverse event
MDSC	Myeloid-derived suppressor cell
NSCLC	Non-small-cell lung cancer
PD-1	Programmed cell death protein 1
PD-L1	Programmed death-ligand 1
PFS	Progression-free survival
RCC	Renal cell carcinoma
ROS	Reactive oxygen species
SCFA	Short-chain fatty acid
TMAO	Trimethylamine N-oxide
VNP20009	Attenuated *Salmonella Typhimurium* strain VNP20009
